# *De novo *sequence assembly of *Albugo candida *reveals a small genome relative to other biotrophic oomycetes

**DOI:** 10.1186/1471-2164-12-503

**Published:** 2011-10-13

**Authors:** Matthew G Links, Eric Holub, Rays HY Jiang, Andrew G Sharpe, Dwayne Hegedus, Elena Beynon, Dean Sillito, Wayne E Clarke, Shihomi Uzuhashi, Mohammad H Borhan

**Affiliations:** 1 Agriculture and Agri-Food Canada, 107 Science Place, Saskatoon, SK., S7N 0X2 Canada; 2 Department of Veterinary Microbiology, 52 Campus Drive, University of Saskatchewan, SK S7N 5B4, Canada; 3 School of Life Sciences, University of Warwick, Wellesbourne campus, Wellesbourne, Warwick, CV35 9EF, UK; 4 The Broad Institute of MIT and Harvard, Massachusetts 02141, USA; 5 National Research Council of Canada - Plant Biotechnology Institute, 110 Gymnasium Place, Saskatoon, SK S7N 0W9 Canada; 6 Canadian Intellectual Property Office, Industry Canada, 50 Victoria Street, Gatineau QC, K1A 0C9, Canada; 7 Department of Computer Science, 110 Science Place, University of Saskatchewan, Saskatoon, SK S7N 5C9, Canada

## Abstract

**Background:**

*Albugo candida *is a biotrophic oomycete that parasitizes various species of Brassicaceae, causing a disease (white blister rust) with remarkable convergence in behaviour to unrelated rusts of basidiomycete fungi.

**Results:**

A recent genome analysis of the oomycete *Hyaloperonospora arabidopsidis *suggests that a reduction in the number of genes encoding secreted pathogenicity proteins, enzymes for assimilation of inorganic nitrogen and sulphur represent a genomic signature for the evolution of obligate biotrophy. Here, we report a draft reference genome of a major crop pathogen *Albugo candida *(another obligate biotrophic oomycete) with an estimated genome of 45.3 Mb. This is very similar to the genome size of a necrotrophic oomycete *Pythium ultimum *(43 Mb) but less than half that of *H. arabidopsidis *(99 Mb). Sequencing of *A. candida *transcripts from infected host tissue and zoosporangia combined with genome-wide annotation revealed 15,824 predicted genes. Most of the predicted genes lack significant similarity with sequences from other oomycetes. Most intriguingly, *A. candida *appears to have a much smaller repertoire of pathogenicity-related proteins than *H. arabidopsidis *including genes that encode RXLR effector proteins, CRINKLER-like genes, and elicitins. Necrosis and Ethylene inducing Peptides were not detected in the genome of *A. candida*. Putative orthologs of tat-C, a component of the twin arginine translocase system, were identified from multiple oomycete genera along with proteins containing putative tat-secretion signal peptides.

**Conclusion:**

*Albugo candida *has a comparatively small genome amongst oomycetes, retains motility of sporangial inoculum, and harbours a much smaller repertoire of candidate effectors than was recently reported for *H. arabidopsidis*. This minimal gene repertoire could indicate a lack of expansion, rather than a reduction, in the number of genes that signify the evolution of biotrophy in oomycetes.

## Background

Oomycetes are a group of eukaryotic micro-organisms of the kingdom Stramenopila that exhibit a wide breadth of life styles from free-living saprophytes in aquatic and soil environments, to above ground endophytes and obligately biotrophic parasites of plants and animals [1]. Despite having filamentous, fungal-like morphology during most of their life cycle, oomycetes are most closely related to brown algae and unicellular diatoms [1-3].

Terrestrial oomycetes cause some of the most economically destructive plant diseases worldwide such as late blight of potato (*Phytophthora infestans*), downy mildews and root rots in a wide range of seed and forage crops, fruits, vegetables and ornamentals. *Albugo candida *(Pers.) Roussel is a key species for comparative genomics in the oomycetes as the archetypal crop pathogen in the Albuginales, which is an order that consists exclusively of obligate biotrophs. The Albuginales diverged early from the Peronosporales, which includes a wider spectrum of necrotrophs (e.g. *Pythium ultimum*), hemibiotrophs (e.g. *P. infestans *and *P. sojae*) and obligate biotrophic downy mildews (e.g. *Hyaloperonospora arabidopsidis*) [4].

Disease resistance to oomycete pathogens has been a major target of plant breeding programs, and also a focus of genetics research to reveal the molecular basis of major resistance genes for use in crops [5-7]. Parallel progress in oomycete genetics has been much slower. However, recent advances from map-based strategies have identified several avirulence (Avr) proteins in downy mildew and *Phytophthora *pathogens which match major disease resistance genes in their natural hosts [8-11]. Advances have also been made in comparative oomycete genomics with public release of reference genomes for three *Phytophthora *species (*P. infestans, P. sojae *and *P. ramorum*), *Pythium ultimum *and *H. arabidopsidis *from large sequencing consortia [12-15], and production of expressed sequence tags (ESTs) for these species [16,17].

Most oomycete Avr proteins share a conserved amino-terminal signal peptide and a specific 'RXLR' motif, which is generally thought to be required for delivery of proteins with inherent virulence functions into the host cell [18-20]. The RXLR motif and flanking amino acids were used to develop algorithms that have enabled identification of more than 370 candidate effector proteins encoded by the genomes of different *Phytophthora *species [21] and 134 in *H. arabidopsidis*, the downy mildew pathogen of *Arabidopsis thaliana *[14]. Mutational and transient expression analyses have confirmed that several *Phytophthora *proteins have a virulence effector function in plants [22-24].

*Albugo *species cause a destructive disease called white blister rust [25,26]. The disease derives its name from the appearance of white pustules, due to enzymatic digestion of epidermal cell wall, on the surface of leaves and other aerial parts of the host [27]. The pustules contain masses of dehydrated sporangiospores that upon re-hydration in water droplets release 4-6 zoospores that can swim to stomatal openings, encyst and produce a germ tube which will extend into the sub-stomatal chamber and penetrate the host cell. A primary vesicle forms in the host cell, which enables further development of intercellular hyphae in a susceptible interaction [28,29]. Stomatal infection and spore dispersal via epidermal emergence indicate convergence in life cycle adaptation to unrelated basidiomycete rust fungi.

White blister rust has been an important experimental model for investigating the molecular basis of disease resistance, pathogen virulence, host-parasite speciation, and the phenomenon of sustained defence suppression that is typically associated with 'green islands' of compatible host tissue [4,6,28,30,31]. For instance, the type species, *Albugo candida *(Pers.) Roussel was originally collected from a wild species *Capsella bursa-pastoris *[32] but represents a highly variable complex of physiological races, each of which are pathogens in different oilseed, vegetable brassicas or other wild crop relatives [33-36]. More narrowly specialized species occur on other wild members of the Brassicaceae such as a common pathogen of *A. thaliana *that was recently named as *Albugo laibachii *[37]. Rare accessions of *A. thaliana *are susceptible under laboratory conditions to *A. candida *races 2 and 7, derived from *Brassica juncea *and *Brassica rapa*, respectively, and have thus enabled genetic characterisation of white rust resistance (*WRR*) genes in a non-host crop relative [6,38]. The first example, *WRR4*, encodes a TIR-NBS-LRR protein which confers broad spectrum resistance to at least four *A. candida *races in *A. thaliana *[6], and also confers resistance in transgenic oilseed crops of *B. juncea *and *B. napus *[39]. The broad spectrum (race non-specific) nature of *WRR4 *suggests that the multiple physiological races of *A. candida *from domestic and wild host species express a highly conserved avirulence protein.

To support efforts for the genetic improvement of white rust control in *Brassica *crops, we have developed genomic resources for *A. candida *to enable identification of candidate effector proteins expressed by the pathogen, including the predicted avirulence protein recognised by *WRR4 *in *A. thaliana*. Here we report analyses of ESTs from two cDNA libraries and a draft genome sequence generated from the *A. candida *race 2 isolate [40] re-designated Ac2VRR (the 'RR' suffix was added to the pathotype name Ac2v to indicate the standard isolate chosen from the collection of the late Dr. Roger Rimmer). The first cDNA library was constructed from susceptible *B. juncea *tissue infected with Ac2VRR, and a second library was made from Ac2VRR sporangiospores collected from susceptible seedlings.

## Results and discussion

### EST production and assembly

A cDNA library was constructed from the susceptible *B. juncea *cultivar 'Cutlass' infected with the *A. candida *race 2 isolate Ac2VRR. Approximately 36,000 cDNA clones (average insert size of 800 bp) were sequenced from both directions and after sequence quality assessment a total of 50,248 ESTs were collected. A computational pipeline was developed to distinguish plant-derived versus pathogen-derived ESTs. ESTs with significant sequence similarity to any plant gene were predicted as being transcripts derived from *B. juncea *during infection, whereas the remaining ESTs were putatively ascribed as originating from the pathogen. This *in silico *separation was intentionally designed to be conservative in assigning a "plant" designation to ESTs, as we sought to be highly confident about identifying sequences that were expressed by the pathogen. As a result, 28% of the assembled cDNAs (14,510) were predicted as being derived from Ac2VRR.

To provide a companion dataset of Ac2VRR transcripts, we also sequenced a full length normalized cDNA library generated from Ac2VRR sporangiospores. These ESTs provide a rich sample of transcripts expressed in the pathogen inoculum, and include genes encoding proteins important for initiating the infection process. A total of 38,704 high quality ESTs were obtained. We compared ESTs from the two complementary libraries to derive a combined data set of 14,376 pathogen transcripts from Ac2VRR, consisting of 2,847 that were present in both libraries, 5,424 that were only found in the library from infected host tissue, and 11,799 from the second 'pre-infection' library generated from sporangiospores (Additional file 1: Additional Table S1).

### Genome sequencing, assembly, and annotation

To validate the predicted *A. candida *cDNAs and to map the physical arrangement of genes within the genome, we generated a draft reference genome by sequencing genomic DNA from sporangiospores of Ac2VRR. Using pyrosequencing we completed a 20 × shotgun coverage of the Ac2VRR genome which included a 7 kb paired-end library (Additional file 1: Additional Table S2). Assembly of the shotgun data yielded 33.9 Mbps in 2,359 contigs of at least 500 bps with an N50 of 77 kb. After scaffolding the assembly was resolved to 252 scaffolds covering 34.5 Mbps with an N50 of 375 kb. Average coverage of the assembled data was identified by performing a reference assembly of the sequencing data against the assembled sequence using Newbler (454/Roche). An average coverage depth of 20 × was observed in the reference assembly and the estimated genome size was calculated by taking the total number of base pairs input to the assembly (919,675,861 bps) and dividing that by the average coverage (20 ×) to yield an estimated genome size of 45.3 Mb which is very close to the previously published estimate of 45.6 Mb [41]. Therefore the scaffolded 34.5 Mb assembly represents 76% of the estimated genome size which compares favourably to the 82-84% of the *H. arabidopsidis *genome recently reported [14]. This disparity between the assembled and estimated genome size could be due to un-resolved repetitive elements or arise from telomeric, centromeric and peri-centromeric regions of the genome which would be inaccessible to these sequencing methods.

Additional validation of the assembly was carried out by alignment of 5 BACs from Ac2VRR which were sequenced as a 7 kb paired-end library using titanium chemistry. Each of the 5 BACs was individually assembled using Newbler and in all 5 cases the data assembled into a single scaffold (Additional file 1: Additional Table S3). The assembled BAC sequences were then aligned to the genomic scaffolds using MUMMER [42] and visually inspected (Additional file 1: Additional Figures S1-S5). The regions of the shotgun genome assembly which correspond to these BACs show a very high level of sequence similarity with the genomic assembly.

The assembled genome had a GC content of approximately 43% with a very minor increase in the coding regions of 1-2%. Repeat structure analysis using RepeatModeler [43] and RepeatMasker [44] revealed that 17% of the assembled genome was repetitive (5% LINEs, 6% LTRs, 3% DNA elements, and 3% Unclassified repeats). Analysis of the single copy Core Eukaryotic Genes (CEGs) via CEGMA [45] revealed that the *A. candida *genome assembly harbours (88%) CEGs as complete (215/248 CEGs) or partial models (additional 2 CEGs). By contrast to the CEGMA analysis, mapping assembled cDNAs using BLAT [46] onto the assembled genome revealed that 98% of the expressed genes were identified within the assembled genome. Therefore while the CEGMA results might imply less than complete genome our assessment of the assembly using the cDNA data demonstrate that this assembly is a *High-Quality Draft *assembly of the *A. candida *gene-space as defined by Chain et al [47]. *De novo *gene prediction was accomplished using software, glimmerHMM [48] and GENEID [49,50], trained specifically for *A. candida *using our cDNA data. Combining multiple types of evidence through the use of Evidence Modeler [51] we identified a total of 15,824 gene models, implying an average inter-genic distance of 2,862 bp. From the alignments of our assembled cDNAs to the genome sequence, it was apparent that the majority (68%) of predicted genes in Ac2VRR do not contain an intron. Validation of the predicted genes in the Ac2VRR genome was achieved using RNA-Seq data from sporangiospores. RNA-Seq data was aligned to the genome using Bowtie [52], TopHat [53], and Cufflinks [54]. The results of the Cufflinks report on the specificity and sensitivity of the gene predictions (Additional file 1: Additional Table S4) indicate a very high accuracy in the prediction of genes within the *Albugo candida *genome with minor discrepancies at intron/exon junctions.

Using the Ac2VRR draft genome, we screened ESTs from the pathogen infected plant material and calculated the number of False Positives, True Positives, False Negatives and True Negatives for our *in silico *separation of host and pathogen ESTs (Additional file 1: Additional Table S5). As expected, this method provided a very high specificity (99%) with low sensitivity (54%). The low sensitivity arises from the fact that many housekeeping genes from Ac2VRR share some level of sequence similarity with orthologous genes in the host plant. Further validation of *A. candida *transcripts was achieved by PCR amplification from genomic DNA (derived from sporangiospores) of a set of 100 cDNAs assigned computationally as being derived from Ac2VRR (data not shown). The high specificity indicates that ESTs being predicted as originating from the pathogen were confirmed within the genome sequence for Ac2VRR. Taken together, these measures of specificity and sensitivity indicate that this computational approach is useful for identifying expressed genes that are highly specific to the pathogen in the absence of a genome sequence for the pathogen.

These genomic data provide an initial opportunity to assess the genome organisation of *A. candida *and the degree to which orthologous genes appear to have been retained relative to other oomycetes. Using BLAST we identified 5,975 putative orthologs in *P. ultimum*, 5,858 to *P. infestans*, 5,581 to *P. sojae*, 5,592 to *P. ramorum*, and 4,922 to *H. arabidopsidis*. We investigated whether *A. candida *was deficient in the same metabolic pathways as those reported for *H. arabidopsidis *[14]. *Albugo candida *is deficient in the same nitrate, nitrite, and sulphite reductases as *H. arabidopsidis *(Additional file 1: Additional Table S6).

### The *Albugo candida *secretome

We identified a secretome comprised of 929 proteins from the annotated set of *Albugo candida *proteins, using previously established criteria [13] such as the presence of amino-terminal signal peptide, a motif indicating that the encoded protein is likely to be secreted by the pathogen, and lack of additional trans-membrane domains. The Ac2VRR secretome appears to be of about 2/3 the size of the *P. infestans *secretome [55]. In contrast to the extensive family expansions observed in *Phytophthora *genomes, the gene families of the *A. candida *secretome have relatively few members (Additional file 1: Additional Table S7). For example, the largest *A. candida *secreted protein family consists of 6 members, whereas the largest family in *Phytophthora *has hundreds of members. Even in the pathogen *H. arabidopsidis *with a reduced genome, the largest effector family is more than a hundred members [14]. Interestingly the largest secreted gene family in *A. candida *is comprised of six Crinkler-like (CRN) proteins. Many of the gene families within *A. candida *encoded proteins with unknown functions, however as described below, some families resembled proteins with functions previously associated with the elicitation of innate defence in plants and/or compatibility in plant-microbe interactions.

In order to identify the complement of Pathogen Associated Molecular Patterns (PAMPs) and effectors present in *A. candida *relative to other oomycetes we screened the predicted proteins in a manner similar to Baxter *et al. *[14]. In general, the *A. candida *genome harbours fewer genes in each of the major classes of PAMP or effector-like proteins (Additional file 1: Additional Table S8).

**Figure 1:**
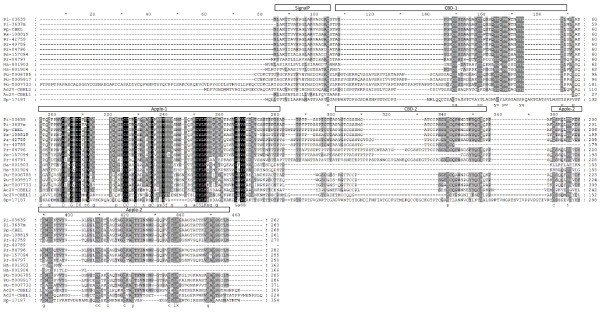
**Alignment of oomycete CBEL proteins**. InterPro domains for *Phytophthora infestans *gene PITG_03639 are indicated above the alignment. Note that the protein sequence for *P. infestans *gene PITG_03637 was manually curated (PITG_3637 m) based on the alignment of PITG_03639 with the genomic sequence of PITG_03637. The common structure of CBELs from plant pathogens is comprised of a N-terminal signal peptide and Cellulose Binding and Apple domains.

Cellulose Binding Elicitor Lectin (CBEL) is a cell wall glycoprotein from *P. parasitica *var *nicotianae *which can elicit host defences in *Nicotiana benthamiana *[56], and includes a family of 13 and 15 genes in *P. sojae *and *P. ramorum*, respectively [14]. Only two genes in the *H. arabidopsidis *genome have been identified as encoding CBELs [14]. The genome of *A. candida *also contains two putative CBEL genes (Additional file 1: Additional Table S9) which were identified based on sequence similarity to CBEL from *P. parasitica *(CAA65843: Pp-CBEL). Ac2VRR-CBEL1 has a signal peptide which may result in its secretion and its expression was detected to be higher during infection than in sporangiospores (Additional file 1: Additional Table S9). By contrast Ac2VRR-CBEL2 does not contain a signal peptide indicating that it would be unlikely to be secreted and its expression was only detected in sporangiospores. Alignment of Ac2VRR-CBEL1, Ac2VRR-CBEL2 with CBEL proteins from other oomycetes indicates that the canonical structure of CBELs from *Phytophthora *species is a sec-dependent secretion signal followed by interleaved Cellulose Binding (CBD) and Apple domains which can mediate protein-protein or protein-carbohydrate interactions (Figure 1). Examining the multiple sequence alignment and the molecular phylogeny of the oomycete CBELs (Additional file 1: Additional Figure S6), variation is observed amongst CBEL orthologs which appears to be conserved within Oomycete genera. Of note is that the CBEL from the fish pathogen *Saprolengia parasitica *has Apple domains in locations which correspond to the location of the CBDs in the plant pathogens. In tests performed using a transient assay in *N. benthamiana *with Ac2VRR-CBEL1, we did not observe necrotic lesions (Additional file 1: Additional Figure S7). As can be seen from Figure 1, Ac2VRR-CBEL1 is missing the corresponding CBD1 domain relative to CBEL from *P. parasitica*. Gaulin and co-workers [57] reported that mutations in any of the aromatic amino acids of either CBD1 or CBD2 disrupted the elicitor activity of Pp-CBEL. Both CBEL genes from *H. arabidopsidis*, another biotrophic oomycete, appear to lack regions corresponding to the second CBD domain and Apple domains of CBEL from *P. parasitica*.

Elicitins belong to PAMPs from plant pathogenic oomycetes. Elicitins are a highly conserved group of secreted proteins and includes members such as INF1, a 10 KDa protein from *P. infestans *that elicits host cell death in *Nicotiana benthamiana *[58-61]. The presence of six cysteine residues is a common feature of elicitins and has been used to mine databases revealing 156 elicitins (ELI) or elicitin-like (ELL) genes from several *Phytophthora *species [58]. Following this precedent, we identified nine genes which were either annotated as elicitins via InterProScan or had similarity to an ELL (Additional file 1: Additional Table S8). Elicitin and elicitin-Like genes were identified as being expressed in both cDNA libraries and were similar to SOJ7, SOJ5 and an ELK gene Ps-7-10d-ZO, from *P. sojae*. Additional sequence similarity for the *A. candida *elicitin-like genes was observed to *P. citrophthora *and INF5 from *P. infestans*.

Necrosis and Ethylene inducing Peptides (NEPs) are common among diverse groups of plant pathogens and are capable of triggering plant cell death [62]. Except *A. candida*, all oomycetes with sequenced genomes have genes encoding NEPs. We performed sequence similarity searches with known NEP proteins and searched the *A. candida *proteome using a HMM which has been used to recover NEP proteins from *Phythophthora *spp. and *H. arabidopsidis*. None of the methods, used to search for NEPs in *A. candida*, yielded any candidate genes.

**Figure 2:**
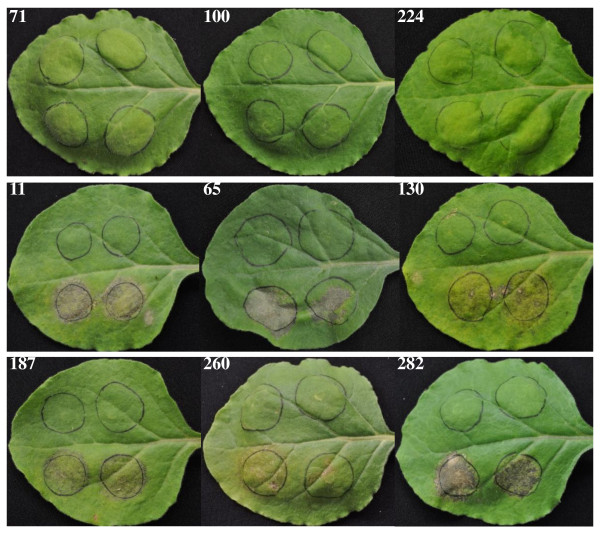
**Transient expression in *Nicotiana benthamiana *leaves of Ac2VRR-RXL candidates from *Albugo candida***. Infiltration of *Agrobacterium tumefaciens *strain GV3101 carrying a pTRBO-Gateway vector expressing Ac2VRR-RXL were performed in duplicate within the tissues marked by circles (two circles below the mid-vein). Control infiltrations were performed using vectors containing GUS in the two upper circles. Numbers on each panel correspond to the Ac2VRR-RXL name in Additional file 1: Additional Table S9.

RXLRs are a group of cytoplasmic effectors that are abundant in *Phytophthora *species [13,21]. *A. candida *contains a small group of genes that encode secreted proteins with a variant RXLR motif (Ac-RXL). The number of Ac-RXL effectors was estimated by string searches within the predicted proteins with amino-terminal signal peptides. The numbers of true positives were derived from subtraction of HMM searches of the permutated protein sequences [21]. We identified a total of 26 predicted gene models which contained a putative sec-dependent signal peptide, lacked homology to known proteins, and had an Ac-RXL motif (Additional file 1: Additional Table S10). The consensus Ac-RXL domain in Ac2VRR appears to be LSSLR(ILKS)L(KQ)SL, based on an analysis of the amino acid conservation in the subset of candidate proteins. Ac2VRR-RXL-65 has an ORF of 450 bps encoding a secreted protein of 17.4 kDa, with low-level expression detected by 1 clone in infected seedling library, and 2 clones in the sporangiospore library. The predicted signal peptide was located at the 18^th ^amino acid (NN Score = 0.72; HMM Score = 0.99). When the candidate Ac2VRR-RXL-65 was transiently expressed in *N. benthamiana *leaves necrosis was visible at the site of infiltration. A series of eight additional candidate Ac-RXLs were tested for their ability to transiently induce necrosis when infiltrated into *N. benthamiana*. Of these additional candidate Ac-RXLs, five were able to induce necrosis in *N. benthamiana *and three were not (Figure 2 and Additional file 1: Additional Table S10). Further investigation is needed to determine the nature of necrosis induced in tobacco and to confirm each effector's function. Conserved sequence modules termed in W-, Y-, L- domains from the C-termini of RXLR proteins in *Phytophthora *and *Hyaloperonospora *have been described [21]. These domains have been shown to be involved in suppression host Program Cell Death [63]. We searched the Ac2VRR proteome with a set of HMM based on W-, Y-, L- domains in the C-termini of RXLR proteins. None of these domains were detected in *Albugo*, which supports the independent origin of *Albugo *effectors in the evolution of biotrophy.

CRNs were first identified in *Phytophthora *species as secreted proteins that have a conserved LFLAK motif downstream of the signal peptide [13]. Homologues have been reported from *H. arabidopsidis *[14], *P. ultimum *[64] and *Aphanomyces euteiches *[65]. CRNs have been proposed to be an ancient group of host-targeting proteins that evolved in the oomycete lineage. Using a modified pattern based on the work of Schornack and co-worker [66] and custom HMMs based on the conserved N-terminal domain containing the host-targeting motif FLAK, we identified six gene models as putative CRNs (Additional file 1: Additional Tables S7 and S8). The host targeting motif LYLAK in *A. candida *is slightly different than canonical CRNs. The *A. candida *CRNs form the largest secreted gene family in *A. candida*. By contrast to *P. sojae*, CRN type effectors are not highly abundant in *A. candida *but are similar in number to *P. ramorum*. Additionally the highest expression level we could detect was a single EST clone for 4 of the 6 *A. candida *CRNs in either of our cDNA libraries. We cloned five of the *Albugo *predicted CRNs and transiently expressed them in *N. benthamiana *leaves. None of the CRN induced any visible response in tobacco.

We systematically searched for novel host-targeting motifs amongst the proteins containing a sec-dependent secretion signal with the program MEME [67], allowing up to 3 motifs to be discovered simultaneously in the N-terminal 30-100 amino acids. One motif, CHxC occurring in several proteins was recovered. The motif showed strong conservation with two Cys residues (Additional file 1: Additional Table S11 and Additional Figure S8). The MEME output for the CHxC motif was subsequently used for a HMM search in *A. candida*. In total, 40 CHxC proteins were found; all of them had no detectable homolog in *Phytophthora *or *Hyaloperonospora *species. The high level of sequence divergence from other species is indicative of their specific adaptation in *Albugo *species.

### Putative Twin Arginine Translocase (tat) secreted proteins

While the sec-dependent secretion system is capable of protein secretion, it is not able to maintain complex protein conformations during secretion [68]. The twin arginine translocation (tat) system, which was first described in plants, appears to be competent for secreting folded proteins and protein complexes [69]. Recent work has indicated that tat-secreted proteins can contribute to virulence in plant pathogens [70]. To date the description of functional tat secretion systems has been limited to bacterial plant pathogens, but there is evidence for the conservation of a component gene of the tat secretion system being present in *P. infestans *(tat-C; GenBank 9695398) [71]. We identified a series of additional tat-C orthologs in the genomes of other oomycetes (Additional file 1: Additional Table S12) by sequence similarity to the published *P. infestans *tat-C. These searches identified putative tat-C orthologs in four oomycete genera (*Phytophthora, Pythium, Hyaloperonospora and Saprolengia*). Tat-C genes are typically encoded within the mitochondrial genome and the sequence of the tat-C gene itself is highly homopolymeric. We could identify a fragment of a tat-C gene within the *A. candida *assembly but full resolution of the gene was not possible given that pyrosequencing data is poorly suited to the resolution of homo-polymeric regions. The tat secretion system recognizes a conserved signal peptide (tat-P) and thus we used an *in silico *analysis of the proteomes of oomycetes to investigate whether tat-P signals were a common feature of the proteins within these organisms (Additional file 1: Additional Table S13). The number of putative tat-P containing proteins in the Ac2VRR genome was much reduced relative to the proteomes of other oomycetes (Additional file 1: Additional Table S14) and the level of conservation of the tat-P motif was low across the sequenced oomycetes (Additional file 1: Additional Figure S9). There were 12 *A. candida *predicted genes that bore similarity to other oomycetes and the fact that there is a level of conservation amongst the oomycetes would suggest there may be a shared evolutionary lineage to some of these genes amongst oomycetes (Additional file 1: Additional Figure S10).

## Conclusions

Here we present a summary of the predicted repertoire of genes from *Albugo candida *using transcript sequences and *de novo *sequence assembly of the pathogen genome. The historical reference isolate of *A. candida *(renamed Ac2VRR) used for this investigation represents a major destructive pathogen in oilseed and vegetable production of brassica crops worldwide. Previous pathology and molecular genetic studies using *A. candida *have been important for advancing our understanding of broad spectrum disease resistance and defence suppression in *A. thaliana *and the genetics of avirulence in *A. candida *[6,30,31].

*Albugo candida *is a member of the Albuginales, which is comprised exclusively of obligate biotrophs that have adapted to a wide diversity of hosts [25,26]. This order is thought to have diverged early in oomycete evolution from other major plant pathogens including necrotrophic *Pythium *species which are the basal lineage in the Peronosporales, with respect to other hemi-biotrophic *Phytophthora *species and obligate biotrophic downy mildews [72]. Thus biotrophy has apparently evolved independently, in two major lineages of the oomycetes. Oomycete genomes range in size from the smallest 18 Mb genome of a necrotrophic *Pythium *[73,74] to the largest > 240 Mb genome of potato late blight pathogen *P. infestans *[13,74]. The *A. candida *genome size (45.3 Mb) is significantly smaller than a comparable biotroph from the Peronosporales such as *H. arabidopsidis*, a common parasite of *A. thaliana *in Europe. Similar to *H. arabidopsidis *[14] and the obligate fungi [75], *A. candida *is deficient for enzymes required for nitrate and sulphate assimilation and has a reduced number of cell wall degrading enzymes.

The most intriguing contrast found in the comparison of *A. candida *genome with other oomycetes is the low number of RXLR effector-like genes (26 were predicted as compared to 563 in *P. infestans*, 350 in the genomes of *P. sojae *and *P. ramorum *and 134 in the genome of *H. arabidopsidis*) [13]. This relatively small gene family in *A. candida *may be due to weak selection pressure from the slow evolution of white rust resistance genes in the host, and/or an apparently minimal repertoire of effectors in *A. candida *that have been more effective in suppressing and avoiding host defences than in members of the Peronosporales. The lack of RXLR genes in the genome of *P. ultimum *[15] and their discovery in a variant (Ac-RXL) form in the *A. candida *genome suggest that this class of effectors may have evolved independently along with biotrophy in the two major lineages of plant pathogenic oomycetes. The potential function of candidate CHxC proteins in *A. candida *also warrants further investigation as a potential novel class of effectors.

A similar conclusion can be drawn from other proteins that have previously been associated with pathogencity in plants. For example, NEPs found in many plant pathogens are capable of inducing cell death in plants [62]. The *P. infestans *and *P. sojae *genomes contain genes that encode 27 and 39 NEPs, respectively. NEP expression levels in *P. sojae *correlates with a transition in disease development from biotrophy to necrotrophy [23]. We were unable to identify any NEP orthologs in *A. candida *from the transcriptome or in the assembled genome sequence. The absence of NEP genes in *A. candida *supports the suggestion of Qutob and co-workers [23] that NEPs are associated with necrotrophy. By contrast to the lack of NEPs in the *A. candida *genome the *H. arabidopsidis *genome contains 10 NEP-like genes. However NEP proteins in *H. arabidopsidis *show high sequence divergence, probably to avoid triggering necrosis response in host [14].

*Albugo candida *and *P. infestans *are also notably different in the respective number of elicitins and crinkler-like proteins encoded in each genome. We identified nine elicitin and elicitin-like genes in Ac2VRR, far fewer than the 156 genes detected in *Phytophthora *species [58]. Crinklers are also highly abundant in *Phytophthora *species with close to 200 genes reported for *P. infestans *[13]. *Phythophthora infestans *crinkler proteins are typified by an amino-terminal LFLAK domain and are capable of inducing necrosis *in planta *[22,66]. While we were able to identify six CRNs in the *A. candida *genome none were detected as being highly expressed during infection or in sporangiospores and they did not induce necrosis in planta.

Presence of tat-C orthologs and the conservation of tat-P containing proteins within the genomes of oomycetes are intriguing. In the case of *Albugo candida *the tat-P containing complement of genes is reduced relative to other oomycetes and the genome appears to harbour a tat-C ortholog. While these observations of a putative tat secretion system within oomycetes are preliminary it forms an intriguing question for further investigation.

The differences in the repertoire of genes involved in pathogencity, between *A. candida *and other oomycetes indicate that *A. candida *is highly adapted to life as an obligate biotroph. Amongst the canonical classes of pathogen effector molecules, many appear to be non-existent in the *A. candida *genome or represent significantly smaller classes of genes. Our analysis of the *A. candida *genome provides a basis for investigating the relationship between an obligate biotroph and its host. In general, these results portray a remarkably small eukaryotic genome for an organism that has evolved intimate biotrophic relationships independently from other oomycete lineages and has adapted to diverse host species.

## Methods

### Plant growth and pathogen inoculation

Seeds of *B. juncea *'Cutlass' (Agriculture and Agri-Food Canada, Saskatoon Research Centre) were planted in a coco-soilless mix and grown in a growth chamber under 16 hrs of light and at 18°C days and 15°C night. Pathogen storage and preparation of inoculum has been described in detail previously [6]. *Albugo candida *sporangiospores were suspended in distilled water (adjusted to ca. 2 × 10^4^) and incubated at 14°C for 2 hrs to facilitate release of zoospores. Seven-days-old seedlings were inoculated on each cotyledon with a drop of the sporangiospores and released zoospore suspensions. Inoculated seedlings were kept in an incubator at 14°C for 24 hrs, and then transferred to the growth chamber. Pustules appeared on the abaxial surface of cotyledons at 7 - 10 days post inoculation (dpi).

### Construction of cDNA libraries and sequencing

To construct the cDNA library from *A. candida *infected plants, RNA was extracted from Cutlass - AC2VRR infected seedlings at 10 dpi, and purified using an mRNA purification kit (GE Healthcare life Science Cat. # 27-9258-02). cDNA was generated from mRNA and cloned into the SalI and NotI sites of pSPORT1, based on instructions provided by the SuperScript cDNA synthesis kit (Invitrogen, Cat. # 18248-013). Approximately 40,000 clones were sequenced from both orientations using T7 and degenerate oligo-dT primers. Sporangiospore cDNA was made from spores collected from heavily diseased seedings of *B. juncea *'Cutlass', at 10 - 14 dpi. A full length cDNA library was made under contract by Vertis Biotechnology AG (Germany). cDNAs were directionally cloned into the EcoRI sites of pJM1 [76]. Sequencing of sporangiospore cDNA clones was carried out in both directions at the University of Washington on commercial contract.

### RNA Sequencing

Sporangiospore mRNA was extracted from spores of heavily diseased seedlings of *B. juncea *'Cutlass', at 10-14 dpi. Total RNA was extracted from sporangiospore using Trizol reagent (Catalogue number 15596-026, Invitrogen) and further purified by Qiagen RNeasy kit (catalogue number 74903, Qiagen) according to the manufacturer's instruction. mRNA was prepared from total RNA using Ambion Poly(A) Purist kit (catalogue number AM1916, Ambion Inc.) based on the instruction provided by the manufacturer. 2 μg mRNA sample was used for sequencing using Illumina platform. RNA-Seq was carried out at the NRC-Plant Biotechnology on an Illumina HiSeq. Data was obtained for 101 cycles of single end sequencing as per the standard Illumina unstranded protocol. RNA-Seq results were de-multiplexed using the Casava 1.8 pipeline (Illumina). Analysis of the RNA-Seq data was achieved using Tophat [53] with parameters modified for the differences between human gene models and those found in *Albugo candida *(-i 20 -I 3000 --min-segment-intron 20 --max-segment-intron 3000). Validation of the gene predictions using the RNA-Seq data was accomplished using Cufflinks [54] (-I 3000 --min-intron-length 20 --max-bundle-length 35000).

### Transient gene expression in *Nicotiana benthamiana*

Candidate effectors were cloned into pSK103 a modified pTRBO [77] vector, a gateway destination vector developed by the laboratory of Dr. Kevin Rozwadowski at the Agriculture and AgriFood Canada Saskatoon Research Centre. Briefly, the predicted ORF of each candidate gene was amplified by PCR using specific primers, with attB1 and attB2 Gateway cloning sites at the 5' of forward and reverse primers, respectively. PCR product was cloned into pDONR-Zeo vector (Invitrogen, Cat. # 12535-035) using BP clonase II enzyme mix (Invitrogen, Cat. # 11789-020). Each DNA fragment was then cloned into pSK103 using LR clonase II enzyme mix from Invitrogen (Cat. # 11791-020). A candidate gene cloned into the psk103-gateway vector was then transferred into *Agrobacterium tumefaciens *strain GV3101 by electroporation. The transformed *A. tumafaciens *lines were grown over night, adjusted to an OD600 of 0.02 in induction media (MES, 10 mM MgCl2 pH5.7, 100 μM Acetosyringone) and used for infiltration into 4 - 6 week old *Nicotiana benthiana *leaves as described previously [77].

### Warehousing and analysis of ESTs

Sequencing trace files were warehoused and handled using APED (http://sourceforge.net/projects/aped). Initial assessments of sequencing efficacy were made by calculating the maximum sustained Phred [78,79] quality value in five successive windows of 20 bps each. This was performed with the reads assessed as Good (sustained quality > 20), Fair (sustained quality between 15 and 20) or Poor (sustained quality < 15). While the initial quality assessment of the reads was based strictly on sequencing efficacy, Lucy [80] was also used to trim each read prior to sequence assembly and to screen the reads for viable cloned products. The assembly of cDNA sequence data was performed using TGICL [81]. Assembled transcript sequence IDs carry a 3-part name delineated by underscores. For example the distinct transcript 681_128_1 indicates that this is a transcript assembled from cDNA project # 681 (Additional file 1: Additional Table S1), cluster 128 and contig 1. Distinct transcripts were annotated via InterPro), after first identifying all putative open reading frames using getORF [82]. Predicted open reading frames shorter than 50 amino acids were excluded from further analysis.

### Genome assembly and annotation

Whole genome shotgun sequencing was carried out for *A. candida *isolate Ac2VRR using DNA extracted from sporangiospores. Spores were ground in pre-chilled mortars; 200 ml of warmed (37°C) extraction buffer (50 mM Tris pH 8.0, 200 mM NaCl, 0.2 mM EDTA, 0.5% sodium dodecyl sulfate, 0.1 mg/ml Proteinase K, 0.1 mg/ml glycogen) was added. Samples were vortexed briefly and incubated at 37°C for 30 minutes. Extractions were carried out with an equal volume of buffer saturated phenol followed by 2 extractions in chloroform:isoamyl alcohol (24:1), with centrifugation of 2 minutes at 800 g for each extraction. Nucleic acid was precipitated using 1/10th of a volume of 3 M sodium acetate (pH 5.2) and 2.5 volumes of cold 95% ethanol and pelleted by centrifugation at 8000 g for 15 minutes at 8°C. Pellets were washed twice with 70% ethanol and re-suspended in 100 ml of TE. Samples were treated with RNAse A (40 mg/mL for 30 minutes at 37°C) and then extracted with equal volumes of phenol:chloroform:isoamyl alcohol (25:24:1) followed by a chloroform extraction. The pellet was stored for an hour at -20°C, precipitated as before, re-suspended in 50-100 mL of 10 mM Tris-Cl, pH 8.5 and stored at 4°C until sequencing. Roche 454 FLX and Titanium shot-gun sequencing was carried out at the NRC-Plant Biotechnology Institute following the procedure as described previously [83] with modifications for the Titanium chemistry as described in protocols supplied by the manufacturer (Roche, Laval, Quebec). Genome assembly was performed using version 2.3 of the Newbler assembly software (Roche/454 Brandford CT, USA). Repeat analysis was performed using RepeatModeler [43] and RepeatMasker [44]. Gene prediction employed the software, glimmerHMM [48] and GENEID [49,50], trained specifically for *A. candida *using our cDNA data. Multiple evidence types for gene prediction were combined using Evidence Modeler [51]. The Ac2VRR genome data has been deposited into the public domain in GenBank (Genome project #56025, SRA submission SRA024984.2).

A Bacterial Artificial Chromosome (BAC) library was constructed from Ac2VRR spores. One week old seedlings of *B. juncea *cultivar cutlass were inoculated with Ac2VRR and spores were collected at 10 to 14 days after inoculation. Library construction was carried out by Bio S&T Inc. (Montreal, Canada) by cloning the high molecular weight DNA into the HindIII site of pIndigoBAC5 (EPICENTRE Biotechnologies; Madison, USA). The library contained total of 3,072 BACs with an average insert size of 160 kb. Five BACs were selected for sequencing to provide external validation of the shotgun genome assembly. For each of the 5 BACs a 7 kb paired-end library was generated and the library sequenced at the NRC-Plant Biotechnology using the Titanium chemistry as described in protocols supplied by the manufacturer (Roche, Laval, Quebec). Sequence data for each BAC was trimmed against the sequences for the pIndigoBAC5 and the *E. coli *K12 genome sequence and assembled using version 2.3 of the Newbler assembly software (Roche/454 Brandford CT, USA).

### Separation of putative *A. candida *ESTs from total cDNA sequence data

To separate the sequence information of pathogen ESTs derived from *B. juncea *infected tissue, we developed a computational pipeline designed to conservatively characterize experimental ESTs as plant-like if they had any significant sequence similarity to a plant sequence database. Using a series of six phases (Additional file 1: Additional Table S15), ESTs were sequentially screened against the BLAST databases using both BLASTN and TBLASTX [84]. ESTs with a significant match (1e-15 cutoff) were removed and putatively ascribed as being plant in origin. The order of the databases within the screening process was designed to facilitate rapid database searches by screening early so as to reduce the number of searches against the increasingly large databases in the later phases of the screening. Assessment of the screening procedure was performed by subsequent comparisons of the ESTs to both the draft genome sequence for *A. candida *Ac2VRR as well as internal *Brassica spp*. datasets. Formal calculation of the efficacy of the pipeline to separate the ESTs was addressed by calculating the Specificity and Sensitivity (Additional file 1: Additional Equation S1, Additional Equation S2). EST sequence data has been submitted to dbEST (HO914811-HO965058, HO965059-HO999999, and HS000001-HS003763).

### Sec-dependent secreted protein annotation

The annotated protein set was subjected to SignalP3.0 prediction [85,86]. A positive candidate is predicted either by the Neural Network or the Hidden Markov Model methods with the cleavage site located between 10 and 30 amino acids in length. Membrane proteins were further separated from the data set if transmembrane domains were predicted in the body of a protein. Proteins targeted to mitochondria or the Endoplasmic Reticulum were removed based on IntroPro annotations and SwissProt protein homology (BLASTP evalue < 1e-5). The final Ac2VRR secretome was found to contain 939 members. Searches for novel host-targeting motifs was carried out using MEME [67] and allowing up to 3 motifs within the first 30-100 amino acids. The protein families were grouped by the program TribeMCL [87], with the parameter I set to be 3.0.

## List of abbreviations

NEP: Necrosis and Ethylene inducing Peptides; Avr: Avirulence; WRR: White Rust Resistence; CWD: Cell Wall Degrading; CBEL: Cellulose Binding Elicitor Lectin; CBD: Celluslose Binding Domain; ELI: Elicitin; ELK: Elicitin Like; HMM: Hidden Markov Model; tat: Twin Arginine Translocase; CRN: Crinkler

## Authors' contributions

MHB directed the research. MGL, MHB, and AGS coordinated the genome sequencing. MGL, MHB, WEC and RHYJ analyzed the data. MHB, MGL, AGS, DH, EBH, RHYJ drafted the manuscript. EB, SU, DS preformed the research. All authors have read and approved the final manuscript.

## Supplementary Material

Additional file 1 **Additional data**. A file containing additional data: 10 additional figures, 16 additional tables and 2 equations. Click here for file
