# Supplementary material for: De novo sequence assembly of Albugo candida reveals a small genome relative to other biotrophic oomycetes
Source: BMC Genomics. 2011 Oct 13;12:503. doi: 10.1186/1471-2164-12-503 (PMC3206522; doi:10.1186/1471-2164-12-503)
Supplement: Additional file 1 — Additional data. A file containing additional data: 10 additional figures, 16 additional tables and 2 equations. [file 1471-2164-12-503-S1.DOC]

**ADDITIONAL DATA**

Additional Figures

Additional Figures S1 to S5

Title: Mummerplot of BACs vs. WGS scaffolds.

Description: Validation of scaffolds by comparison with BACs

Additional Figure S6

Title: Phylogeny of CBEL genes

Description: Phylogeny of CBEL genes from oomycete plant pathogens.

Additional Figure S7

Title: Transient expression of *A. candida* CBEL

Description: Transient expression in *Nicotiana benthamiana* leaves of CBEL transcripts. Infiltrated part of the leaf is marked by a circle. A: CBEL from *Phytophthora parasitica.* B: *Albugo candida* 2VRR CBEL1. C: Control infiltration of vector containing GUS.

Additional Figure S8

Title: Sequence logo of the *A. candida* CHxC domain.

Description: Sequence logo of the Albugo candida CHxC domain. The domain occurs within 100 aa of the N-termini of proteins with a sec-dependent secretion signal. Cys residues are shown by orange letters, the basic residues are coloured blue, acidic residues are coloured red, hydrophobic residues are indicated by blue letters.

Additional Figure S9

Title: Sequence logos of the tat-P sites from oomycetes.

Description: Sequence logos of the amino acids flanking the tat-P sites identified in the respective proteome of *Albugo candida* and five other oomycetes, including a necrotroph (*Pythium ultimum*)*,* three hemibiotrophs (*Phytophthora sojae,* *P. infestans* and *P. ramorum*) and an obligate biotroph (*Hyaloperonospora arabidopsidis*).

Additional Figure S10

Title: Venn diagram of putative tatP secretion signal containing proteins.

Description: Venn diagram showing the *Albugo candida* gene models which contain a putative tatP secretion signal and have a BLAST similarity (1e-5) to a gene in five other oomycetes, including a necrotroph (*Pythium ultimum*)*,* three hemibiotrophs (*Phytophthora sojae,* *P. infestans* and *P. ramorum*) and an obligate biotroph (*Hyaloperonospora arabidopsidis*). In red is shown the two proteins which bear similarity to proteins in *Arabidopsis thaliana*.

**Additional Tables**

Additional Table S1

Title: Summary of gene expression from two cDNA libraries from *A. candida.*

Description: Summary of gene expression from two cDNA libraries including the results of assembly, and the accounting of predicted transcripts from plant (*Brassica juncea* ‘Cutlass’) vs. pathogen (*Albugo candida* race 2, isolate Ac2VRR) following infection of seedlings.

Additional Table S2

Title: Assembly metrics*.*

Description: Assembly metrics for draft assembly of the *Albugo candida* genome*.*

Additional Table S3

Title: Assembly results for BACs*.*

Description: Assembly results of BAC 454 sequencing.

Additional Table S4

Title: RNA-Seq validation of genes.

Description: RNA-Seq validation of predicted genes.

Additional Table S5

Title: Validation for predicting the origin of ESTs.

Description: Results of validation exercise for predicting the origin of ESTs.

Additional Table S6

Title: Presence of biotrophy related genes in *A. candida*

Description: Presence of genes suggested by Baxter et al (2010) as being signatures for obligate biotrophy. Shaded are genes which are absent in H. arabidopsidis or A. candida.

Additional Table S7

Title: Secreted Protein families.

Description: Secreted Protein families identified in *A. candida* by TribeMCL.

Additional Table S8

Title: *A. candida* PAMPs and effectors

Description: Number of A. candida genes for classes of secreted PAMPs and effectors relative to other oomycete genomes using data from Baxter et al. (2010). BLAST similarity was based on a 1e-7 cutoff.

Additional Table S9

Title: Identifier tracking of cDNA assembly IDs.

Description: Identifier tracking of cDNA assembly IDs across libraries and # of clones / transcript for transcripts discussed in manuscript.

Additional Table S10

Title: Ac-RXLs.

Description: Candidate Ac-RXLs

Additional Table S11

Title: *A. candida* CHxC genes.

Description: *A. candida*  genes containing CHxC domains.

Additional Table S12

Title: Putative tat-C orthologues.

Description: Putative tat-C orthologues identified in Oomycete genomes. BLAST results were obtained using the P. infestans tat-C (NP_037620.1) as the query.

Additional Table S13

Title: Regular expression for tatP secreted protein identification.

Description: Regular Expression used to identify possible tatP secreted proteins. The trailing single quote is needed for rendering within Microsoft Word and is not part of the regular expression.

Additional Table S14

Title: Size of the tat-P proteomes.

Description: Size of the tat-P containing subset of the proteomes.

Additional Table S15

Title: Phases of BLAST differentiation to identify origin of ESTs.

Description: Phases of BLAST based screening performed to putatively ascribe ESTs as either plant or pathogen derived.

Additional Table S16

Title: InterPro terms used for functional categories.

Description: InterPro terms used for functional categories in Additional Table 12

**Additional Equations**

Additional Equation 1.

Title: Calculation of Specificity for calling experimentally derived ESTs as putatively plant in origin.

Additional Equation 2.

Title: Calculation of Sensitivity for calling experimentally derived ESTs as putatively pathogen in origin.

**Additional Figures**

**
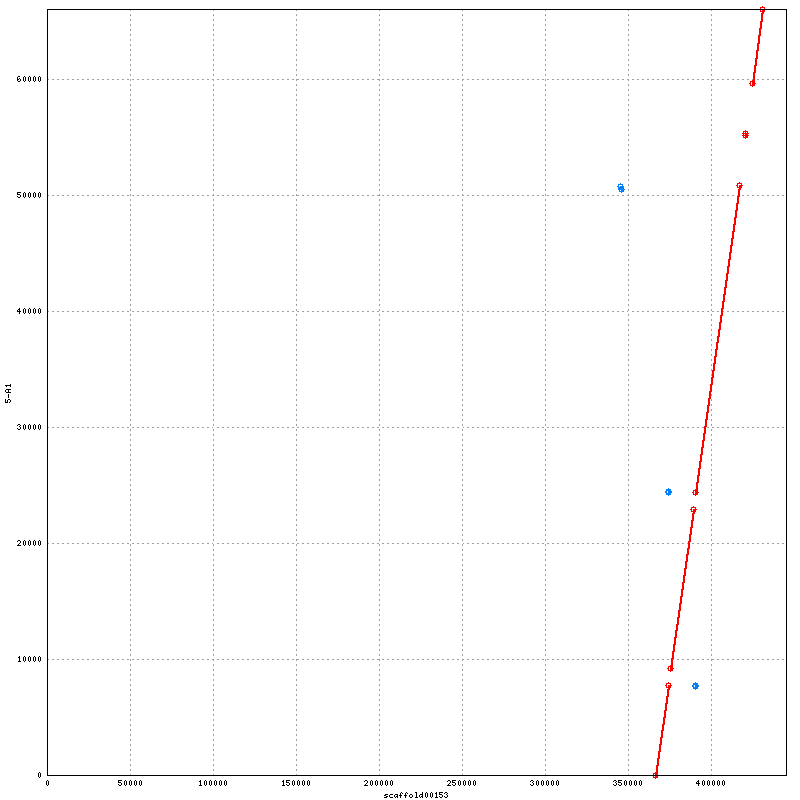
**

Additional Figure S2: Mummerplot of BAC 5-A1 vs. scaffold00153

**
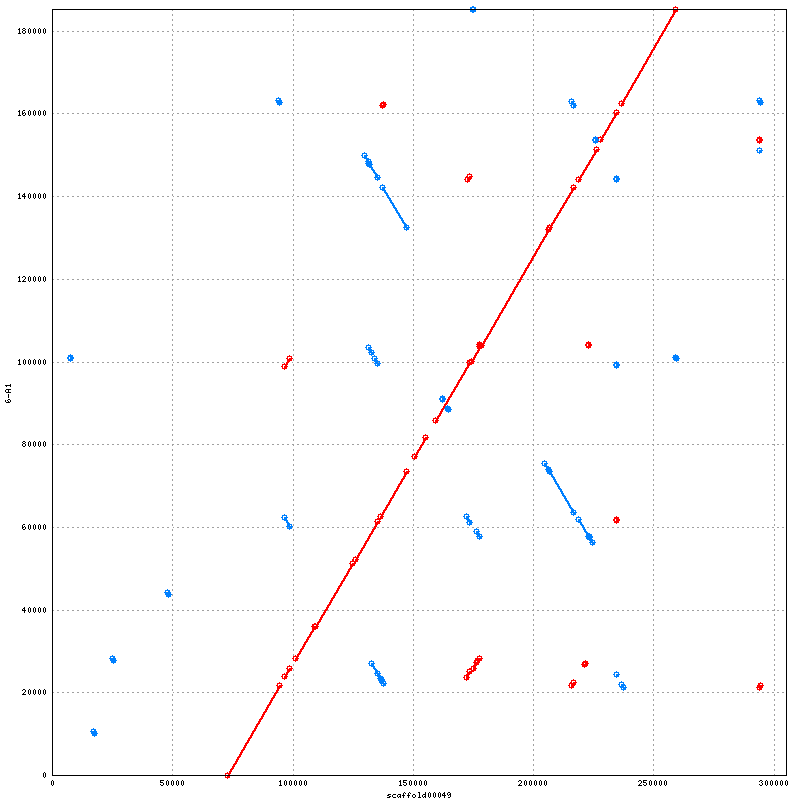
**

Additional Figure S3: Mummerplot of BAC 6-A1 vs. scaffold00049

**
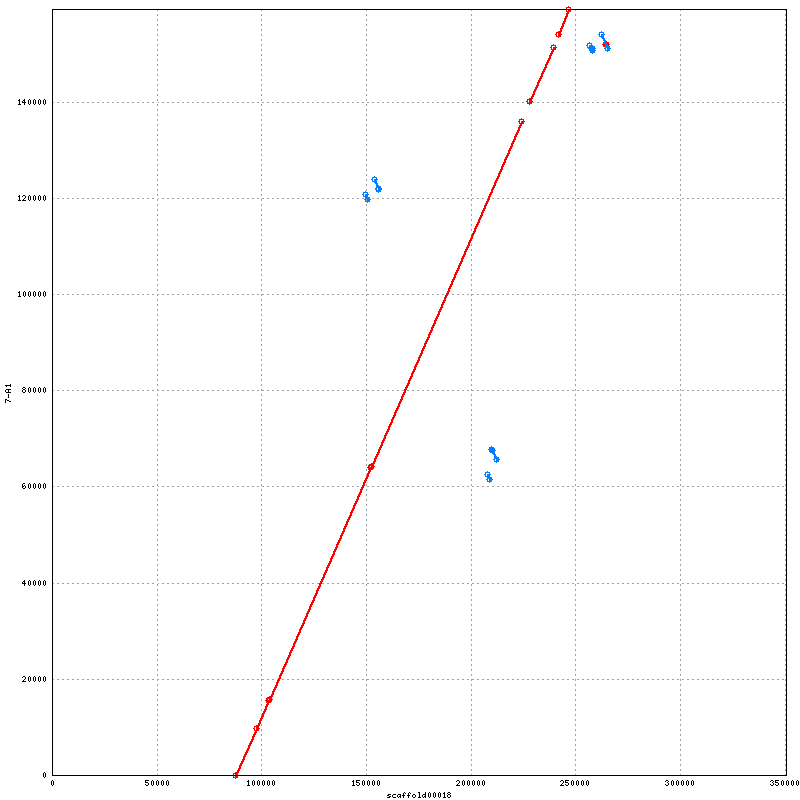
**

Additional Figure S4: Mummerplot of BAC 7-A1 vs. scaffold00018

**
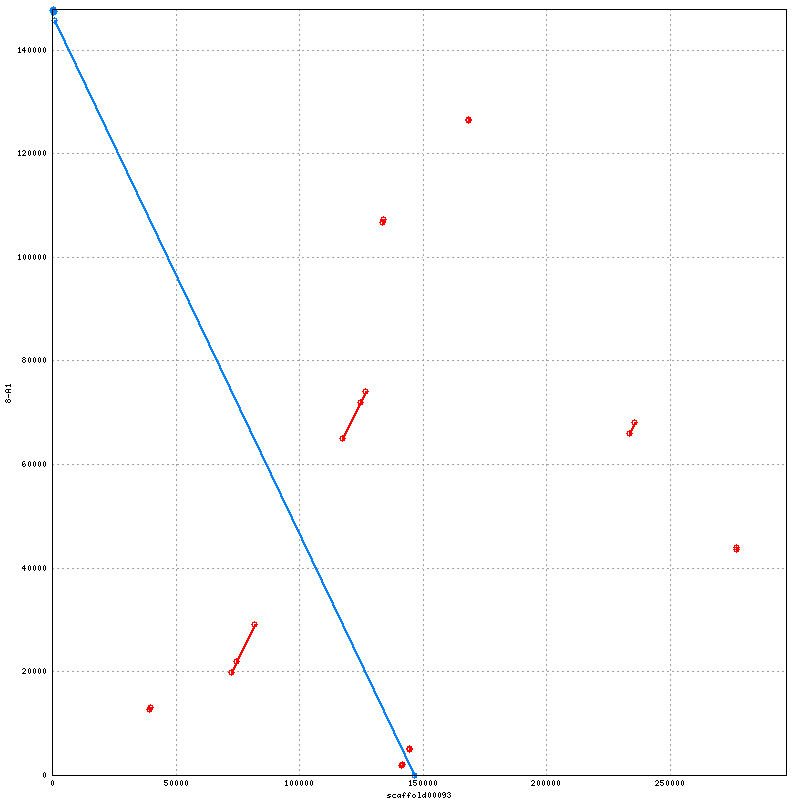
**

Additional Figure S5: Mummerplot of BAC 8-A1 vs. scaffold00093.

**
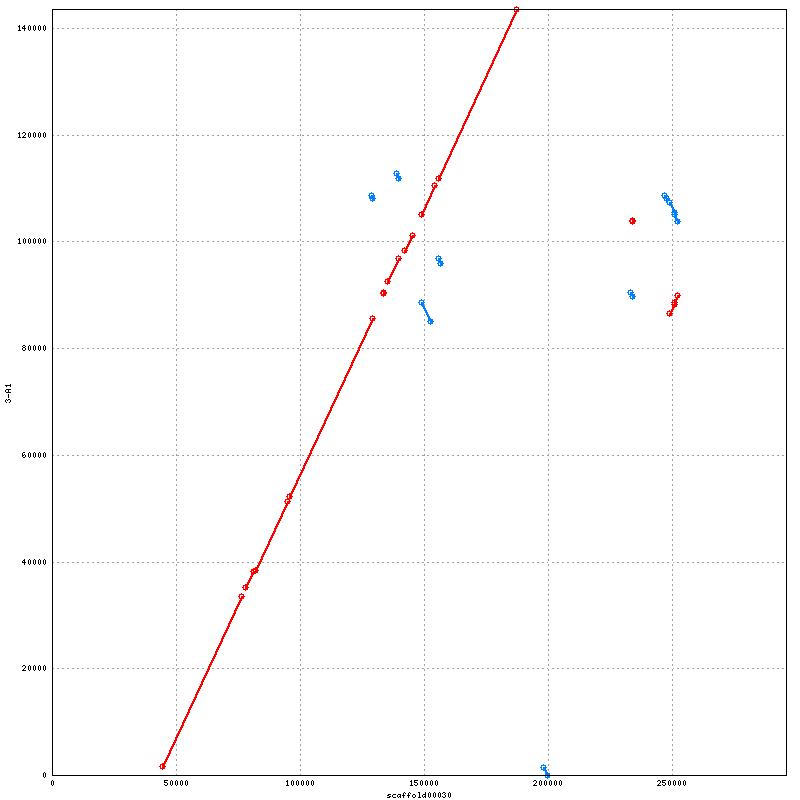
**

Additional Figure S6: Mummerplot of BAC 3-A1 vs. scaffold00030.

**
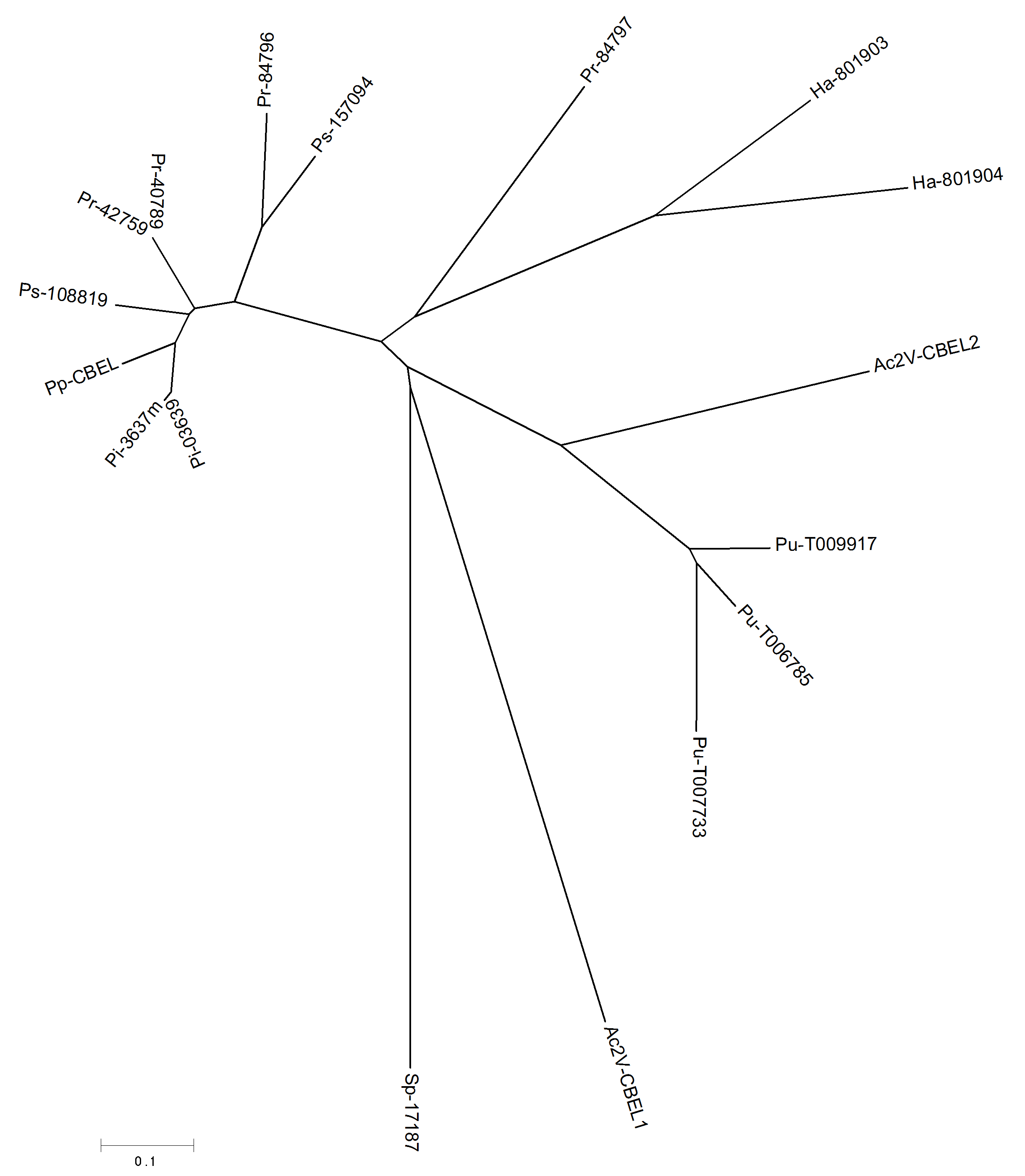
**

Additional Figure S7: Phylogeny of CBEL genes from oomycete plant pathogens. The CBEL from the fish pathogen *Saprolengia parasitica* is included.


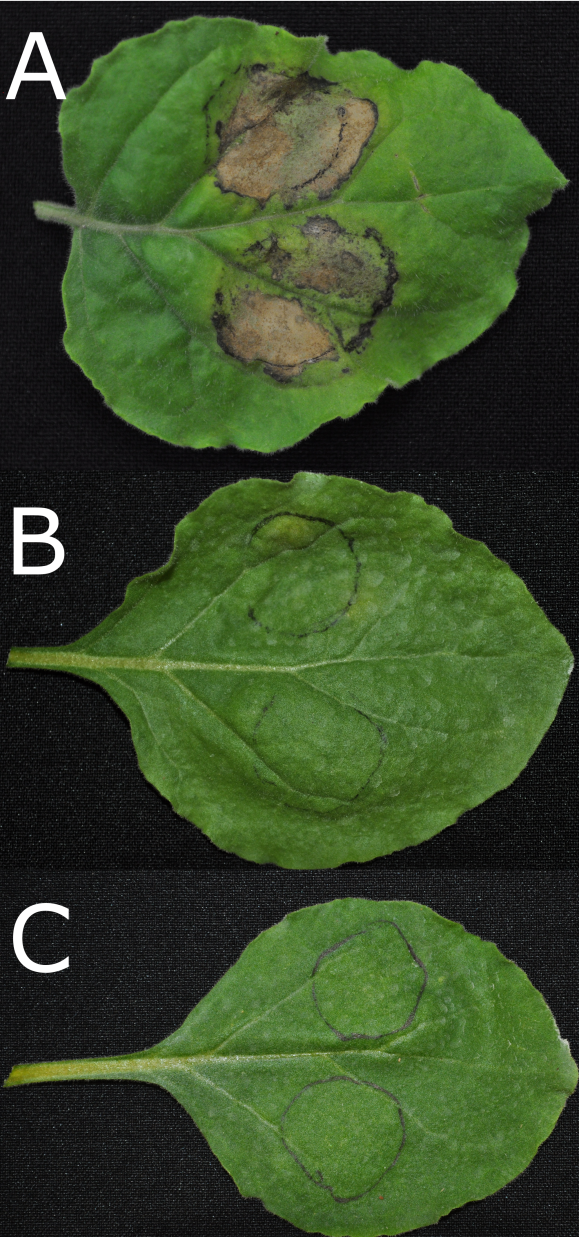


Additional Figure S8: Transient expression in *Nicotiana benthamiana* leaves of CBEL transcripts. Infiltrated part of the leaf is marked by a circle. A: CBEL from *Phytophthora parasitica.* B: *Albugo candida* 2VRR CBEL1. C: Control infiltration of vector containing GUS.


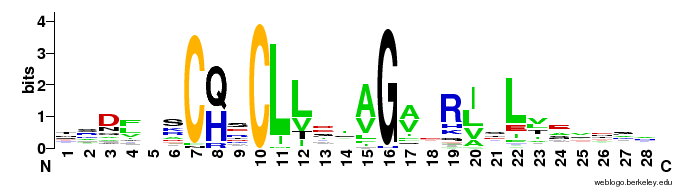


Additional Figure S9: Sequence logo of the *Albugo candida* CHxC domain. The domain occurs within 100 aa of the N-termini of proteins with a sec-dependent secretion signal. Cys residues are shown by orange letters, the basic residues are coloured blue, acidic residues are coloured red, hydrophobic residues are indicated by blue letters.

**
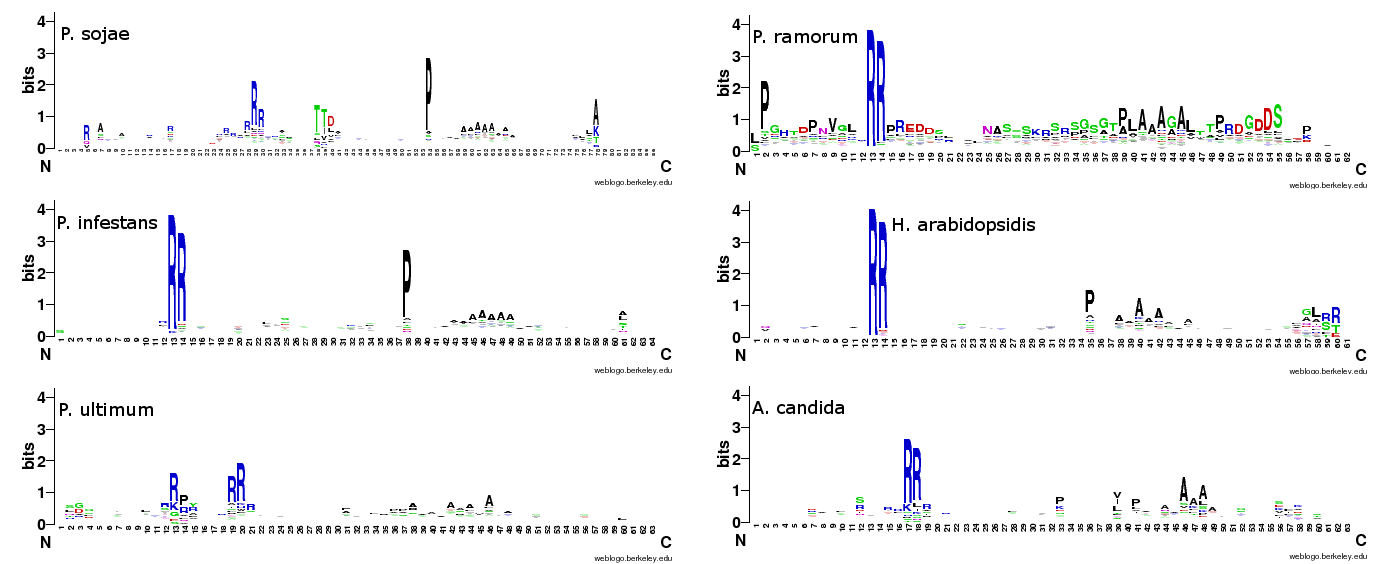
**

Additional Figure S10: Sequence logos of the amino acids flanking the tat-P sites identified in the respective proteome of *Albugo candida* and five other oomycetes, including a necrotroph (*Pythium ultimum*)*,* three hemibiotrophs (*Phytophthora sojae,* *P. infestans* and *P. ramorum*) and an obligate biotroph (*Hyaloperonospora arabidopsidis*).


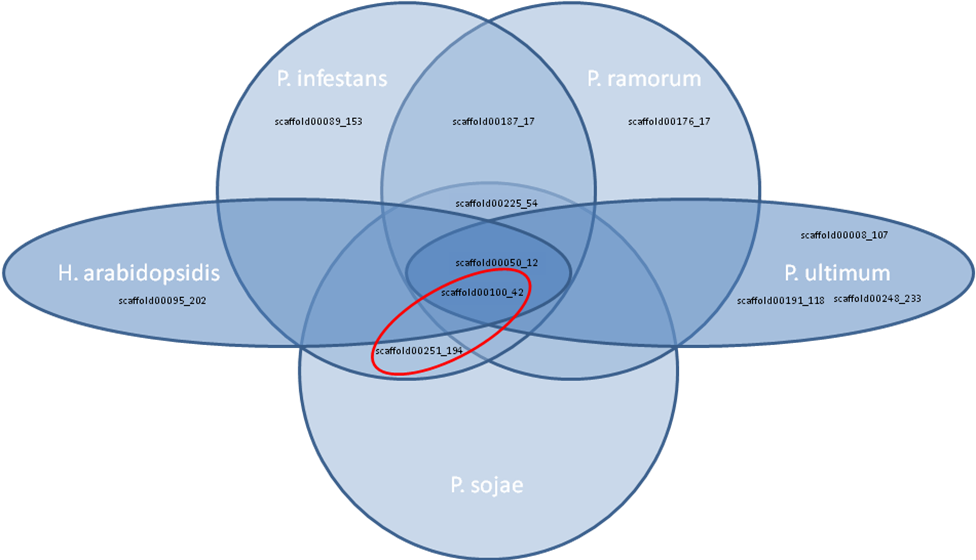


Additional Figure S11: Venn diagram showing the *Albugo candida* gene models which contain a putative tatP secretion signal and have a BLAST similarity (1e-5) to a gene in five other oomycetes, including a necrotroph (*Pythium ultimum*)*,* three hemibiotrophs (*Phytophthora sojae,* *P. infestans* and *P. ramorum*) and an obligate biotroph (*Hyaloperonospora arabidopsidis*). In red is shown the two proteins which bear similarity to proteins in *Arabidopsis thaliana*.

**Additional Tables**

Additional Table S1: Summary of gene expression from two cDNA libraries including the results of assembly, and the accounting of predicted transcripts from plant (*Brassica juncea* ‘Cutlass’) vs. pathogen (*Albugo candida* race 2, isolate Ac2VRR) following infection of seedlings.

|  |  | **Sequencing Efficacy^1^** | | | **cDNA clustering + assembly** | |
| --- | --- | --- | --- | --- | --- | --- |
| **Library description** | **# reads** | **Good** | **Fair** | **Poor** | **Input:**  **# High quality reads** | **Output:**  **# distinct transcripts** |
| Total ESTs derived from infected tissue library (ID-179) | 69,119 | 73.5 | 3.8 | 22.6 | 50,248 | --- |
| Predicted plant ESTs from infected tissue library (ID-333) | 35,738 | --- | --- | --- | 35,738 | 13,346 |
| Predicted pathogen ESTs from infected tissue library (ID-332) | 14,510 | --- | --- | --- | 14,510 | 5,424 |
| Pathogen ESTs from spore library (ID-682) | 73,438 | 52.8 | 6.4 | 40.8 | 38,704 | 11,799 |
| Combined ESTs from infected tissue and spore libraries (ID-681) | 87,948 | 60.6 | 5.3 | 34.1 | 53,214 | 14,376 |

^1^Based on maximum sustained Phred score.

Additional Table S2: Assembly metrics for draft assembly of the *Albugo candida* genome*.*

| **Input** | **Value** |
| --- | --- |
| # reads | 3,307,379 |
| # bps | 919,675,861 |
| Estimated fold coverage | 20x |
|  |  |
| **Scaffolds** | |
| # of scaffolds | 252 |
| # bp | 34,563,972 |
| # Mbp | 34.5 |
| Average scaffold length | 137,158 |
| N50 scaffold size | 375,021 |
| Largest scaffold | 1,097,187 |
|  |  |
| **Large contigs (minimum 500bp)** | |
| # of contigs | 2,359 |
| # bp | 33,922,053 |
| # Mbp | 33.9 |
| Average contig length | 14,379 |
| N50 contig size | 76,763 |
| Largest contig | 422,999 |
|  |  |
| **All contigs (minimum 100bp)** | |
| # of contigs | 5,393 |
| # of bp | 34,628,338 |
| # Mbp | 34.6 |

Additional Table S3: Assembly results of BAC 454 sequencing*.*

|  | Input to assembly | | Scaffolds | | Contigs > 500 bps | | | |
| --- | --- | --- | --- | --- | --- | --- | --- | --- |
| BAC | # reads | # bps | # | bps in total | # | bps in total | mean length | N50 length |
| 5-A1 | 63975 | 11827571 | 1 | 65988 | 5 | 56174 | 11234 | 13660 |
| 6-A1 | 97825 | 19423235 | 1 | 185266 | 7 | 181940 | 25991 | 59056 |
| 7-A1 | 52538 | 11081195 | 1 | 159275 | 1 | 159275 | 159275 | 159275 |
| 8-A1 | 76274 | 13843620 | 1 | 147821 | 3 | 146959 | 48986 | 145755 |
| 3-A1 | 159140 | 35235126 | 1 | 143565 | 14 | 150134 | 10723 | 88973 |

Additional Table S4: RNA-Seq validation of predicted genes.

|  | **Sn** | **Sp** | **fSn** | **fSp** |
| --- | --- | --- | --- | --- |
| **Base level** | 100 | 99.1 | - | - |
| **Exon level** | 93.4 | 92.3 | 93.9 | 92.8 |
| **Intron level** | 100 | 98.3 | 100 | 98.3 |
| **Intron chain level** | 99.6 | 97.9 | 100 | 98.7 |
| **Transcript level** | 90.3 | 89.5 | 91 | 90.2 |
| **Locus level** | 93.2 | 93.5 | 93.7 | 94 |
|  | Frequency | Percentage | |  |
| **Missed exons** | 0/24626 | 0.0% | |  |
| **Wrong exons** | 146/24926 | 0.6% | |  |
| **Missed introns** | 0/8102 | 0.0% | |  |
| **Wrong introns** | 116/8240 | 1.4% | |  |
| **Missed loci** | 0/16524 | 0.0% | |  |
| **Wrong loci** | 44/16481 | 0.3% | |  |

Additional Table S5: Results of validation exercise for predicting the origin of ESTs.

| **Validation results** | |
| --- | --- |
| False Positives | 12,137 |
| True Positives | 23,601 |
| False Negatives | 240 |
| True Negatives | 14,270 |
|  |  |
| Specificity | 99% |
| Sensitivity | 54% |

Additional Table S6: Presence of genes suggested by Baxter et al (2010) as being signatures for obligate biotrophy. Shaded are genes which are absent in *H. arabidopsidis* or *A. candida*.

|  |  | **This paper** | **Baxter et al. 2010 - Table S3** |
| --- | --- | --- | --- |
| **Gene** | **P. infestans** | **Ac2VRR** | **H. arabidopsidis** |
| Nitrate reductase | PITG_13012T0 | None | None |
| Nitrite reductase | PITG_13013T0 | None | None |
| Nitrate transporter | PITG_13011T0 | None | None |
| Glutamine synthetase | PITG_14180T0, PITG_14179T0 | scaffold00216_37 | Ha802420 |
| Glutamate synthase (NADH) | PITG_07380T0 | scaffold00077_54 | Ha805196 |
| Glutamate synthase (Ferridoxin) | PITG_12037T0, PITG_16280T0 | scaffold00060_115 | Ha812981 |
| Glutamate dehydrogenase | PITG_07671T0 | scaffold00136_123 | Ha805610; Ha806617 |
| ATP sulfurylase Adenylsulfate kinase Pyrophosphatase | PITG_04010T0 | scaffold00221_42 | Ha813786 |
| Phosphoadenosine phosphosulfate reductase | PITG_04601T0 | None | Ha809449 |
| Sulfite reductase | PITG_19263T0, PITG_18187T0 | None | None |
| Cysteine synthetase | PITG_12727T0, PITG_12725T0 | scaffold00187_143 | Ha814750 |

Additional Table S7: Secreted Protein families identified in *A. candida* by TribeMCL.

| **Family** | **# genes** | **Annotation** | **Gene ID** |
| --- | --- | --- | --- |
| T_1 | 6 | Crinkler | scaffold00011_58 scaffold00011_43 scaffold00167_112 scaffold00029_13 scaffold00011_64 scaffold00167_85 |
| T_2 | 5 |  | scaffold00095_186 scaffold00221_76 scaffold00049_11 scaffold00106_61 scaffold00191_44 |
| T_3 | 4 |  | scaffold00089_59 scaffold00039_2 scaffold00185_534 scaffold00147_4 |
| T_4 | 4 | Elicitin | scaffold00023_129 scaffold00023_107 scaffold00023_99 scaffold00023_95 |
| T_5 | 4 |  | scaffold00066_67 scaffold00038_93 scaffold00230_67 scaffold00021_12 |
| T_6 | 4 |  | scaffold00054_112 scaffold00113_29 scaffold00025_4 scaffold00026_1 |
| T_7 | 4 |  | scaffold00167_34 scaffold00175_3 scaffold00185_22 scaffold00215_85 |
| T_8 | 4 |  | scaffold00095_57 scaffold00095_58 scaffold00237_23 scaffold00023_68 |
| T_9 | 3 | Peptidase_C69 | scaffold00085_208 scaffold00085_210 scaffold00085_194 |
| T_10 | 3 |  | scaffold00168_72 scaffold00168_66 scaffold00168_63 |
| T_11 | 3 |  | scaffold00169_19 scaffold00169_38 scaffold00169_27 |
| T_12 | 3 |  | scaffold00153_197 scaffold00153_186 scaffold00153_178 |
| T_13 | 3 |  | scaffold00093_61 scaffold00093_29 scaffold00093_43 |
| T_14 | 3 |  | scaffold00224_282 scaffold00224_278 scaffold00224_262 |
| T_15 | 3 | Glyco_hydro_3 | scaffold00227_105 scaffold00227_104 scaffold00227_100 |
| T_16 | 3 |  | scaffold00153_110 scaffold00153_102 scaffold00153_112 |
| T_17 | 3 |  | scaffold00216_56 scaffold00216_59 scaffold00216_60 |
| T_18 | 3 |  | scaffold00009_52 scaffold00009_63 scaffold00009_55 |
| T_19 | 2 |  | scaffold00149_88 scaffold00185_365 |
| T_20 | 2 |  | scaffold00102_19 scaffold00201_3 |
| T_21 | 2 | Cellulase | scaffold00036_20 scaffold00036_22 |
| T_22 | 2 |  | scaffold00102_7 scaffold00201_15 |
| T_23 | 2 |  | scaffold00217_17 scaffold00184_15 |
| T_24 | 2 |  | scaffold00061_103 scaffold00185_538 |
| T_25 | 2 |  | scaffold00225_12 scaffold00092_14 |
| T_26 | 2 | Glyco_hydro_28 | scaffold00143_42 scaffold00143_81 |
| T_27 | 2 |  | scaffold00221_64 scaffold00221_62 |
| T_28 | 2 | Glyco_hydro_32C | scaffold00224_113 scaffold00224_151 |
| T_29 | 2 |  | scaffold00071_33 scaffold00251_270 |
| T_30 | 2 |  | scaffold00009_5 scaffold00049_40 |
| T_31 | 2 | DUF1191 | scaffold00040_17 scaffold00136_138 |
| T_32 | 2 |  | scaffold00162_64 scaffold00162_53 |
| T_33 | 2 |  | scaffold00232_213 scaffold00232_210 |
| T_34 | 2 | Glyco_hydro_31 | scaffold00038_56 scaffold00038_39 |
| T_35 | 2 |  | scaffold00061_58 scaffold00061_83 |
| T_36 | 2 |  | scaffold00077_73 scaffold00077_72 |
| T_37 | 2 |  | scaffold00183_12 scaffold00185_494 |
| T_38 | 2 | LSM | scaffold00038_112 scaffold00038_119 |
| T_39 | 2 |  | scaffold00243_37 scaffold00243_38 |
| T_40 | 2 |  | scaffold00039_73 scaffold00039_66 |
| T_41 | 2 |  | scaffold00039_71 scaffold00039_64 |
| T_42 | 2 |  | scaffold00143_62 scaffold00143_80 |
| T_43 | 2 | Asp | scaffold00085_262 scaffold00085_318 |
| T_44 | 2 | DnaJ | scaffold00176_146 scaffold00002_11 |
| T_45 | 2 |  | scaffold00201_48 scaffold00201_50 |
| T_46 | 2 |  | scaffold00230_32 scaffold00230_34 |
| T_47 | 2 |  | scaffold00215_46 scaffold00214_18 |
| T_48 | 2 |  | scaffold00154_12 scaffold00098_94 |
| T_49 | 2 |  | scaffold00032_51 scaffold00232_19 |
| T_50 | 2 |  | scaffold00216_67 scaffold00216_69 |
| T_51 | 2 | Elicitin | scaffold00130_64 scaffold00023_85 |
| T_52 | 2 | Lipase_3 | scaffold00032_85 scaffold00032_150 |
| T_53 | 2 |  | scaffold00196_1 scaffold00216_96 |

Additional Table S8: Number of *A. candida* genes for classes of secreted PAMPs and effectors relative to other oomycete genomes using data from Baxter et al. (2010). BLAST similarity was based on a 1e-7 cutoff.

| Gene product | *H. arabidopsidis* | *P. sojae* | *P. ramorum* | *A. candida 2V* | Basis for identification in Ac2V | |
| --- | --- | --- | --- | --- | --- | --- |
| Glycosyl hydrolases | >60 | 125 | 114 | 13 | InterPro terms | 72 terms (Additional Table 13) |
| Cutinases | 2 | 16 | 4 | 0 |  | IPR002200, IPR000675 |
| Chitinases | 1 | 5 | 2 | 3 |  | 14 (Additional Table 13) |
| Endoglucanases | 3 | 10 | 8 | 0 |  | IPR000334 |
| Elicitins | 1 | 18 | 17 | 4 |  | IPR002200 |
| Elicitin-like (but not Elicitin by InterPro) | 14 | 39 | 31 | 5 | Similarity  (1e-7 cutoff) | *P. sojae:* SOL7, SOJ5, SOL11B, and AAO24658; *P. citrophora:* ABH11748; *P. ramorum* RAL11B |
| CBEL and CBEL-like | 2 | 13 | 15 | 1 |  | *P. infestans:* ACM68430 |
| Crinklers | 20 | 40 | 8 | 6 | Pattern recognition | Custom HMM |
| RXLR / Ac-RXL | 134 | 396 | 374 | 26 |  | LSSLR(ILKS)L(KQ)SL |

Additional Table S9: Identifier tracking of cDNA assembly IDs across libraries and # of clones / transcript for transcripts discussed in manuscript.

| **Gene discussed in manuscript** | **Gene Model ID** | **Transcript ID** | **Infected library** | **# clones in the Infected Library** | **Spore library** | **# clones in Spore Library** | **Relative abundance Log2(Infected / Sporangiospore)** |
| --- | --- | --- | --- | --- | --- | --- | --- |
| Ac2VRR-CELLULASE1 | scaffold00012_53 | 681_3686_1 | 332_2007_1 | 2 | 682_6601_1 | 1 | 1.0 |
| Ac2VRR-CELLULASE2 | scaffold00251_169 | 681_2734_1 | 332_426_1 | 4 | N/D | 0 | unique to infected |
| Ac2VRR-CELLULASE3 | scaffold00046_8 | 681_460_2 | Single clone: 2VP30_D10 | 1 | 682_798_1 | 5 | -2.3 |
| Ac2VRR-CBEL1 | scaffold00230_175 | 681_21_1 | 332_19_1 | 25 | 682_3039_1 | 1 | 4.6 |
| Ac2VRR-CBEL2 | scaffold00153_191 | 681_150_1 | N/D | 0 | 682_42_1 | 9 | unique to sporangiospore |
| Ac2VRR-RXL-65 | scaffold00100_47 | 681_1896_1 | Single clone: 2VP20_C14 | 1 | 682_1576_1 | 2 | -1.0 |

Additional Table S10: Candidate Ac-RXLs

| **ID** | **SignalP cleavage site (aa)** | **Size**  **(aa)** | **RXLX position (aa)** | **RXLX (underlined) and flanking region** | | | **Selection criteria*** | | **Clone** | | **HR** | **Alternative name** | | |  |
| --- | --- | --- | --- | --- | --- | --- | --- | --- | --- | --- | --- | --- | --- | --- | --- |
| scaffold00002_17 | 21 | 119 | 57_63 | SNHPSQRSLRSAVVGSADDNA | | | a,b,c,e | |  | |  |  | | |  |
| scaffold00003_45 | 17 | 192 | 51_57 | SRQFVTRILGEELEKTQLYVT | | | a,b,d,e | |  | |  |  | | |  |
| scaffold00018_98 | 17 | 218 | 30_36 | RYEQEVRSLFPDTTIVSVPPI | | | a,b,d,e | |  | |  |  | | |  |
| scaffold00023_26 | 28 | 106 | 35_41 | GFVKLIRRLRNSSECETAAFN | | | a,b,c | |  | |  |  | | |  |
| scaffold00023_68 | 27 | 145 | 43_49 | HGSVRYRELRIAEQRNSNYEI | | | a,b,c,e,f | | 2VP37_G20 | | + | Ac2VRR-RXL-130 | | |  |
| scaffold00038_220 | 27 | 363 | 43_49 | PSTVPQRGLRVGIQLNTQNER | | | a,b,c,e | | 2VP31_L03 | | - | Ac2VRR-RXL-100 | | |  |
| scaffold00060_5 | 23 | 211 | 77_83 | PSENVVRHLRVVVESIQDQVK | | | a,b,c,e | |  | |  |  | | |  |
| scaffold00071_68 | 16 | 307 | 64_70 | SHFRIVRGLRRHNPTIKCVDK | | | a,b,c,e | |  | |  |  | | |  |
| scaffold00077_67 | 26 | 126 | 65_71 | ALLERRRRLRNLHLEPNSDEF | | | a,b,c | |  | |  |  | | |  |
| scaffold00095_219 | 26 | 117 | 39_45 | DGSGAKRSLRKSPLLSLNRPR | | | a,b,c | |  | |  |  | | |  |
| scaffold00100_47 | 19 | 151 | 44_50 | FNINNCRSLKQDESDRQENLR | | | a,b,d,e,f | | 2VP20_C14 | | + | Ac2VRR-RXL-65 | | |  |
| scaffold00113_19 | 25 | 287 | 67_73 | KLIATSRHLRKCVVPLFEDLQ | | | a,b,c | |  | |  |  | | |  |
| scaffold00141_29 | 23 | 180 | 32_38 | YPDSSLRQLKRGDVERAEPGM | | | a,b,d,e | | 2VP60_P04 | | - | Ac2VRR-RXL-224 | | |  |
| scaffold00141_74 | 21 | 323 | 79_85 | QNLIEQRSLQPAIDCRNCSFA | | | a,b,d,e | |  | |  |  | | |  |
| scaffold00141_78 | 24 | 92 | 73_79 | AVQRLERSLIECKDCGFDTST | | | a,b,d | |  | |  |  | | |  |
| scaffold00141_86 | 28 | 349 | 79_85 | HVRTSRRFLGASNRDFERNLA | | | a,b,d,e | | 2VP24_G07 | | - | Ac2VRR-RXL-71 | | |  |
| scaffold00153_110 | 19 | 323 | 65_71 | SLDIEPRHLRHSTSEAVLQIK | | | a,b,c | |  | |  |  | | |  |
| scaffold00153_112 | 19 | 319 | 65_71 | SLNIEPRHLRDNTNEAVLQIK | | | a,b,c | |  | |  |  | | |  |
| scaffold00185_244 | 22 | 298 | 53_59 | FRSTLNRKLRAGSSNIHTTIP | | | a,b,c,e | |  | |  |  | | |  |
| scaffold00201_106 | 20 | 565 | 76_82 | TVTASWRALQRVNEYCAETFN | | | a,b,d,e,f | | 2VP35_P23 | | + | Ac2VRR-RXL-282 | | |  |
| scaffold00201_111 | 25 | 578 | 80_86 | SITGSLRALQRVQDSYCVRDP | | | a,b,d,e | |  | |  |  | | |  |
| scaffold00208_43 | 33 | 101 | 79_85 | SINGWCRHLRCEKKRLKFQAC | | | a,b,d,e | |  | |  |  | | |  |
| scaffold00232_88 | 23 | 504 | 33_39 | TPKKNRRVLKCRDCILSNFHG | | | a,b,d,e | |  | |  |  | | |  |
| scaffold00037_151 | 20 | 165 | 54_60 | EVPSQNRILMESDDDNSYITH | | | a,b,d,e,f | | 2VP6_B17 | | + | Ac2VRR-RXL-11 | | |  |
| scaffold00056_11 | 20 | 264 | 67_73 | CNHNTRRTLADIIARTIEGKY | | | a,b,d,e,f | | 2VP69_A18 | | + | Ac2VRR-RXL-260 | | |  |
| scaffold00037_290 | 20 | 167 | 22_28 | NASNSIRSLTSIEIDVELPWG | | | a,b,d,e,f | | 2VP50_K15 | | + | Ac2VRR-RXL-187 | | |  |
|  |  |  |  | |  |  | |  | |  | | |  |  | |
| *A combination of selection criteria have been used based on RXLR effector features | | | | | | | | | | | | | | | |
| a | presence of N-terminal signal peptide | | | | | | | | | | | | | | |
| b | lack of homology to known sequneces | | | | | | | | | | | | | | |
| c | occurrence of RXLR motif in the N-terminal | | | | | | | | | | | | | | |
| d | occurrence of RXLX motif in the N-terminal | | | | | | | | | | | | | | |
| e | having EST to support the expression | | | | | | | | | | | | | | |
| f | experimental verification | | | | | | | | | | | | | | |

Additional Table S11: *A. candida*  genes containing CHxC domains.

| Gene ID | HMM score* | E value | Position (aa) | CHxC domain |  |
| --- | --- | --- | --- | --- | --- |
| scaffold00098_1 | 41.5 | 1.60E-09 | 41-68 | ARNTKACHSCLIEKVGVERIHLVAINED |  |
| scaffold00093_61 | 43.7 | 3.60E-10 | 41-68 | DKDVMSCHSCLLKIVGVERVLLVANEAP |  |
| scaffold00093_43 | 41.4 | 1.70E-09 | 41-68 | DKNVMTCHSCLLQMVGVERIVLVANEAP |  |
| scaffold00093_29 | 42.3 | 9.00E-10 | 41-68 | DRNDMSCHSCLLQMVGVERIVLVASEAQ |  |
| scaffold00215_36 | 36.2 | 6.20E-08 | 37-64 | DSDGSSCHECLVISAGFQKIYLRQVGYR |  |
| scaffold00150_121 | 19.7 | 0.0032 | 50-74 | ---FRRCQTCLLDIAGVSRVSLLSTNHG |  |
| scaffold00071_26 | 35.3 | 1.20E-07 | 37-64 | HPSVQDCRTCLLKEAGATRLILVEKTIT |  |
| scaffold00232_213 | 31.5 | 1.60E-06 | 39-66 | KSKLNSCQGCLLSQVGALRAKLTLQKNG |  |
| scaffold00232_210 | 36 | 7.30E-08 | 39-66 | KYDLLSCQRCLLSQVGALRAKLTLRENG |  |
| scaffold00201_48 | 35.6 | 1.60E-08 | 40-67 | LPEMCSCQNCILNVAGAMRATIIHMDKA |  |
| scaffold00092_7 | 11.7 | 0.03 | 31-57 | LPYISRCQECLLH-AGAIHVEEEEVPVN |  |
| scaffold00154_12 | 30.6 | 3.20E-06 | 32-59 | MEAYEACQKCLLERAGAIHVEEVNEIGN |  |
| scaffold00012_46 | 36.3 | 6.00E-08 | 32-59 | NAYIDSCQMCLLERGGATHIQWVEVLSE |  |
| scaffold00189_96 | 36.6 | 4.80E-08 | 35-62 | NRDLSGCHECLAVSVGVKELCLLESNSR |  |
| scaffold00232_88 | 33.2 | 5.20E-07 | 33-60 | NRRVLKCRDCILSNFHGDNITLIREGAH |  |
| scaffold00201_15 | 37.9 | 2.00E-08 | 34-61 | NSDLGKCQSCLTDIAGMDRLSLASPPTD |  |
| scaffold00102_7 | 32.3 | 9.40E-07 | 34-61 | NSNLGKCQSCLTDIVGTDPLLLTSPPTH |  |
| scaffold00169_19 | 33.2 | 5.00E-07 | 37-64 | PGDFTRCHECLVSTLGSAQIHLLATKSA |  |
| scaffold00169_27 | 34.2 | 2.50E-07 | 37-64 | PGDFTRCHECLVSTLGSAQIHLLATNSA |  |
| scaffold00187_12 | 18.5 | 0.0013 | 37-64 | RDAFHDCQLCLLSKAGVVKLRITEPRSD |  |
| scaffold00040_14 | 13.9 | 0.0075 | 35-62 | REQYSRCQHCMVIKAGVSGISLDEVDHH |  |
| scaffold00025_4 | 36 | 7.00E-08 | 37-64 | RQAFYLCHDCLVSSFGIEQIRLITATNA |  |
| scaffold00093_51 | 43.6 | 3.90E-10 | 41-68 | RRNAKRCRSCLIQIVGVDRITLVEATSH |  |
| scaffold00074_1 | 34.6 | 1.90E-07 | 32-59 | SAVSENCQKCLLECAGAIHVEEIEETRR |  |
| scaffold00169_38 | 36.8 | 4.00E-08 | 37-64 | SGDFTSCHECLIGTLGATQLHLLVANSA |  |
| scaffold00098_94 | 36.2 | 6.60E-08 | 32-59 | SKVSESCQQCLLERAGAFHVEEVNEFQK |  |
| scaffold00008_1 | 41.4 | 1.70E-09 | 40-67 | SQDHASCHQCLVISAGFEKICLLQVKNK |  |
| scaffold00061_103 | 33.1 | 9.20E-08 | 40-67 | SVDHAACHECLINSLGCAKLYLLQVGEM |  |
| scaffold00071_138 | 44.8 | 1.60E-10 | 41-68 | TAQVEDCHTCLLRNVGVERITLVASNKI |  |
| scaffold00248_95 | 35 | 1.50E-07 | 32-59 | TKNFEVCQRCLLEDAGALFVEEYGKDTR |  |
| scaffold00054_112 | 42.2 | 9.80E-10 | 41-68 | TRDFAACHRCLVLCIGIERIDLIEASKA |  |
| scaffold00113_29 | 38.9 | 9.70E-09 | 35-62 | TRSFSACHRCLVLSIGIKRIDLIEVSKD |  |
| scaffold00201_3 | 35.6 | 9.50E-08 | 33-60 | TSDLGECQSCLTDIAGLDRLSFTSSPVH |  |
| scaffold00102_19 | 35.9 | 7.60E-08 | 41-68 | TSDLGKCQSCLTDIAGVNRLSFAPSFVH |  |
| scaffold00201_37 | 39.8 | 5.30E-09 | 37-64 | TSTLKKCQTCLLEQAGATRLILMKKTIT |  |
| scaffold00188_19 | 13.4 | 0.0091 | 43-70 | VAVYKSFQRCLLDVAGVYRVSITSDSSR |  |
| scaffold00201_22 | 33.8 | 3.30E-07 | 34-61 | VSDLVKCQSCLTDIAGLNRLSLAHIESI |  |
| scaffold00023_139 | 5.9 | 0.32 | 40-67 | YHQFHNCQKCLLIAAGIRKFSITESALF |  |
| scaffold00201_58 | 35 | 1.40E-07 | 42-69 | YKDVIECQDCILKVAGAMRATRYRGDAS |  |
| scaffold00201_50 | 27.2 | 3.20E-05 | 40-67 | YLQILLNQRCILRVAGAMRATVSNVEKA |  |
| *HMM was constructed with the de novo motif search out from the program MEME. | | | | | |

Additional Table S12: Putative tat-C orthologues identified in Oomycete genomes. BLAST results were obtained using the *P. infestans* tat-C (NP_037620.1) as the query.

| Oomycete | Accession | Score | Query coverage | E value |
| --- | --- | --- | --- | --- |
| *P. infestans* | NP_037620.1 | N/A | N/A | N/A |
| *P. andina* | YP_004564323.1 | 429 | 100% | 1.00E-156 |
| *P. mirabilis* | YP_004563956.1 | 426 | 100% | 2.00E-155 |
| *P. phaseoli* | YP_004564257.1 | 278 | 99% | 9.00E-97 |
| *P. sojae* | YP_001165409.1 | 229 | 88% | 7.00E-78 |
| *P. ramorum* | YP_001165362.1 | 224 | 88% | 7.00E-76 |
| *P. ultimum* | YP_003734828.1 | 199 | 82% | 8.00E-66 |
| *S. ferax* | YP_052894.1 | 104 | 87% | 4.00E-29 |
| *H. arabidopsidis* | scaffold_927:1523-2281^*^ | 399 | 86% | 1.00E-111 |

**H. arabidopsidis* tat-C was identified in scaffold 927 at position 1523-2281 from the genome assembly version 8.3

Additional Table S13: Regular Expression used to identify possible tatP secreted proteins. The trailing single quote is needed for rendering within Microsoft Word and is not part of the regular expression.

| **Pattern name** | **Regular expression** |
| --- | --- |
| tatP | ^.{50,150}\w{10}RR\w{10,20}P\w{2,10}A.A\w{10}' |

Additional Table S14: Size of the tat-P containing subset of the proteomes.

| **Organism** | **# putative tatP containing proteins** | **# of proteins in genome** |
| --- | --- | --- |
| *P. sojae* | 360 | 19027 |
| *P. ramorum* | 286 | 15743 |
| *P. infestans* | 189 | 18138 |
| *A. thaliana* | 169 | 27379 |
| *H. arabidopsidis* | 146 | 15511 |
| *P. ultimum* | 143 | 15323 |
| *A. candida* | 54 | 15824 |

Additional Table S15: Phases of BLAST based screening performed to putatively ascribe ESTs as either plant or pathogen derived.

| **BLAST databases** |
| --- |
| Phase 1: In house Brassica data (Aug 29/07) |
| Phase 2: Public Brassica data downloaded from GenBank PLN (Aug 28/07) |
| Phase 3: PlantANNOT database downloaded from TAIR (Aug 29/07) |
| Phase 4: TAIR 7 Arabidopsis release (Apr 20/07) |
| Phase 5: Brassica oleracea data downloaded from TIGR (Jan 30/07) |
| Phase 6: PlantEST database downloaded from GenBank dbEST (Aug 30/07) |

Additional Table S16: InterPro terms used for functional categories in Additional Table 12

| **IPR** | **Used to define** |
| --- | --- |
| IPR018232 | Glycosyl hydrolases |
| IPR001661 | Glycosyl hydrolases |
| IPR023099 | Glycosyl hydrolases |
| IPR000322 | Glycosyl hydrolases |
| IPR004199 | Glycosyl hydrolases |
| IPR006101 | Glycosyl hydrolases |
| IPR006102 | Glycosyl hydrolases |
| IPR006103 | Glycosyl hydrolases |
| IPR001860 | Glycosyl hydrolases |
| IPR000556 | Glycosyl hydrolases |
| IPR000490 | Glycosyl hydrolases |
| IPR002196 | Glycosyl hydrolases |
| IPR002241 | Glycosyl hydrolases |
| IPR002252 | Glycosyl hydrolases |
| IPR004867 | Glycosyl hydrolases |
| IPR001137 | Glycosyl hydrolases |
| IPR001139 | Glycosyl hydrolases |
| IPR001382 | Glycosyl hydrolases |
| IPR001701 | Glycosyl hydrolases |
| IPR000334 | Glycosyl hydrolases |
| IPR002772 | Glycosyl hydrolases |
| IPR000400 | Glycosyl hydrolases |
| IPR001764 | Glycosyl hydrolases |
| IPR001722 | Glycosyl hydrolases |
| IPR002594 | Glycosyl hydrolases |
| IPR000974 | Glycosyl hydrolases |
| IPR001000 | Glycosyl hydrolases |
| IPR005201 | Glycosyl hydrolases |
| IPR000805 | Glycosyl hydrolases |
| IPR002037 | Glycosyl hydrolases |
| IPR002053 | Glycosyl hydrolases |
| IPR000726 | Glycosyl hydrolases |
| IPR001360 | Glycosyl hydrolases |
| IPR001362 | Glycosyl hydrolases |
| IPR006710 | Glycosyl hydrolases |
| IPR000757 | Glycosyl hydrolases |
| IPR000743 | Glycosyl hydrolases |
| IPR001540 | Glycosyl hydrolases |
| IPR005193 | Glycosyl hydrolases |
| IPR000165 | Glycosyl hydrolases |
| IPR001524 | Glycosyl hydrolases |
| IPR005192 | Glycosyl hydrolases |
| IPR005199 | Glycosyl hydrolases |
| IPR005200 | Glycosyl hydrolases |
| IPR001088 | Glycosyl hydrolases |
| IPR001286 | Glycosyl hydrolases |
| IPR000602 | Glycosyl hydrolases |
| IPR000514 | Glycosyl hydrolases |
| IPR001944 | Glycosyl hydrolases |
| IPR001547 | Glycosyl hydrolases |
| IPR001554 | Glycosyl hydrolases |
| IPR000852 | Glycosyl hydrolases |
| IPR018221 | Glycosyl hydrolases |
| IPR013812 | Glycosyl hydrolases |
| IPR008263 | Glycosyl hydrolases |
| IPR008270 | Glycosyl hydrolases |
| IPR011613 | Glycosyl hydrolases |
| IPR018087 | Glycosyl hydrolases |
| IPR013319 | Glycosyl hydrolases |
| IPR006104 | Glycosyl hydrolases |
| IPR018238 | Glycosyl hydrolases |
| IPR016840 | Glycosyl hydrolases |
| IPR016283 | Glycosyl hydrolases |
| IPR017736 | Glycosyl hydrolases |
| IPR019800 | Glycosyl hydrolases |
| IPR019801 | Glycosyl hydrolases |
| IPR019802 | Glycosyl hydrolases |
| IPR006046 | Glycosyl hydrolases |
| IPR023232 | Glycosyl hydrolases |
| IPR004300 | Glycosyl hydrolases |
| IPR005197 | Glycosyl hydrolases |
| IPR005198 | Glycosyl hydrolases |
| IPR001916 | Glycosyl hydrolases |
| IPR011150 | Cutinases |
| IPR000675 | Cutinases |
| IPR000334 | Endoglucanases |
| IPR016283 | Chitinases |
| IPR011583 | Chitinases |
| IPR013540 | Chitinases |
| IPR000726 | Chitinases |
| IPR003610 | Chitinases |
| IPR001579 | Chitinases |
| IPR018371 | Chitinases |
| IPR005089 | Chitinases |
| IPR022409 | Chitinases |
| IPR001002 | Chitinases |
| IPR001223 | Chitinases |
| IPR009470 | Chitinases |
| IPR000070 | Chitinases |
| IPR012334 | Chitinases |
| IPR018040 | Chitinases |

**Additional Equations**

Additional Equation S3: Calculation of Specificity for calling experimentally derived ESTs as putatively plant in origin.

$$Specificity=\frac{TrueNegatives}{TrueNegatives+FalsePositives}$$

Additional Equation S4: Calculation of Sensitivity for calling experimentally derived ESTs as putatively pathogen in origin.

$$Sensitivity=\frac{TruePositives}{TruePositives+FalseNegatives}$$
